# Bridging quantum noise and classical electrodynamics with stochastic methods

**DOI:** 10.1038/s41467-026-73066-4

**Published:** 2026-05-19

**Authors:** Felix Hitzelhammer, Johannes Stowasser, Lukas Hanschke, Katarina Boos, Tobias C. Sutter, Michael Haider, Christian Jirauschek, Kai Müller, Gabriela Slavcheva, Ulrich Hohenester

**Affiliations:** 1 Institute of Physics, NAWI Graz, University of Graz, Graz, Austria; 2 TUM School of Computation, Information and Technology, Technical University of Munich, Garching, Germany; 3 Walter Schottky Institut, Technical University of Munich, Garching, Germany; 4 Faculty of Physics, University of Vienna, Vienna, Austria; 5 Professorship of Electromagnetic Theory and Photonics, University of the Bundeswehr Munich, Neubiberg, Germany; 6 TUM Center for Quantum Engineering (ZQE), Garching, Germany; 7 Munich Center for Quantum Science and Technology (MCQST), Munich, Germany; 8 Quantopticon, Chicago, IL USA

**Keywords:** Computational science, Optics and photonics, Quantum simulation, Quantum physics

## Abstract

The development of emerging technologies in quantum optics demands accurate models that faithfully capture genuine quantum effects. Mature semiclassical approaches reach their limits when confronted with quantized electromagnetic fields, while full Hilbert space treatments are often computationally prohibitive. To address these challenges, we develop a framework based on coupled stochastic processes with a common cross-covariance structure that can be easily coupled to various types of Maxwell solvers. Our approach accounts for the non-commutativity in the quantum-to-classical transition in a natural way, and has the ability to capture quantum optical signatures while retaining compatibility with classical electromagnetics. For benchmarking, we compare our simulation results with experimental emission spectra of a strongly driven InGaAs quantum dot, finding excellent agreement. Our results highlight the potential of tailored stochastic processes for simulating non-classical light in complex photonic environments.

## Abstract (web summary)

Simulation methods for light-matter interactions able to reproduce quantum phenomena are typically either too idealised or too computationally expensive, and cannot be easily interfaced with classical Maxwell solvers. Here, the authors propose and numerically test a modelling framework for light-matter interactions using stochastic differential equations, which can properly account for the dynamics of open quantum systems.

## Introduction

Modeling light–matter interactions on the nanoscale across different architectures in quantum photonics is essential for advancing the design and fabrication of the key components of many emerging quantum technologies^1–5^. Prominent examples include quantum communications, photonic quantum computing, and high-precision quantum sensing^6–9^. Classical electromagnetic solvers based on Maxwell’s equations provide a powerful framework for simulating complex nanostructures^10–12^, but they are inherently restricted to classical light propagation and cannot capture non-classical features such as photon correlations or entanglement. These phenomena require quantum simulation approaches, typically formulated through (quantum) master equations or related quantum-optical models implemented on classical computers. However, these models often describe idealized systems and cannot account for propagation and quantum interference effects in realistic extended macroscopic devices and circuits with different materials, geometries, and boundary conditions. The latter is of particular importance for the design and optimization of the key components of quantum-photonic technologies, e.g., quantum emitters, quantum memories, and repeaters, etc. There is currently a lack of design, modeling, and simulation tools for quantum photonics that would allow predicting and optimizing the quantum performance of such realistic devices. Achieving peak quantum performance of the individual quantum-photonic components is paramount for the realization of the quantum photonic integrated circuits on a chip. Integrated architectures and mass production of consistently highly-performant components, by common consent, represent the way forward to achieving scalability. A unified approach that bridges quantum and classical descriptions is therefore highly desirable not solely from a long-standing foundational point of view^13–15^ but also for the practical realization of quantum technologies.

Quantum descriptions such as the Gorini-Kossakowski-Sudarshan-Lindblad formalism^16,17^ or quantum stochastic differential equations^18^ are elegant and fundamental. However, they do not provide access to the spatial and temporal properties of the electromagnetic field, nor do they offer a natural route for coupling to classical Maxwell solvers. One could instead rely on quantum electrodynamics methods, which quantize the electromagnetic field, but these approaches can quickly become computationally prohibitive for large or complex systems due to the exponential growth of the Hilbert space^19,20^. Furthermore, semiclassical approximations that allow coupling to electromagnetic solvers, e.g., based on the Maxwell-Bloch equations^21,22^ or more advanced Maxwell-Bloch Langevin approaches^23–26^, must be used with caution, as they cannot accurately capture quantum features such as non-classical photon statistics and correlations. This motivates the use of stochastic differential equations (SDEs), which are intrinsically driven by noise, to capture correlations, fluctuations, and noise spectra beyond average dynamics.

The derivation of suitable SDEs from the underlying quantum dynamics employs phase-space representations^27–29^, quantum trajectory methods^30–32^, or quantum Langevin equations^33,34^. Phase-space methods, such as the Wigner or positive *P* representations, map the density matrix onto a quasi-probability distribution, which is governed by a Fokker–Planck equation (FPE). By extracting drift and diffusion from this FPE, one obtains the corresponding SDE^35–38^. Quantum trajectory methods, also known as Monte Carlo wave-function approaches, describe the system in terms of individual stochastic realizations of its wavefunction. They naturally incorporate the randomness associated with quantum jumps, continuous monitoring, and measurement back-action^30–32^. Quantum Langevin equations offer an operator-level description of system-bath interactions. Under suitable approximations, such as linearization or semiclassical limits, they can be recast as SDEs for classical variables (c-numbers)^14,33,34,39^. Here, we introduce an SDE approach, without relying on wave-function unraveling, phase-space representations, or operator-valued noise.

As a first validation of our approach, we consider the Mollow spectrum, which serves as a compelling example of the successful interplay between theory and experiment^40,41^. It arises from the resonance fluorescence of a strongly driven two-level system (TLS)^42^, exhibiting a characteristic triplet structure that reflects coherent light–matter interactions. Excitonic transitions in solid-state systems, such as semiconductor quantum dots (QDs), can be modeled as effective TLSs, and the associated Mollow spectrum has been experimentally observed^43,44^. The Mollow spectrum not only benchmarks theoretical models but also illustrates the importance of capturing fluctuations and correlations in open quantum systems^45^. Beyond providing a benchmark for theoretical models, the sidebands in resonance fluorescence can exhibit non-classical photon correlations, highlighting the relevance of the Mollow triplet for quantum-optical control and photon-correlation experiments^46,47^. The Mollow triplet emission provides a promising approach for single-photon and sequential photon generation^48^, as well as for light–matter entangled-state generation^44,49^.

Motivated by the incompatibility between quantum simulations and Maxwell solvers, we introduce a stochastic Bloch-vector approach underpinned by SDEs, and validate it by comparison to dedicated experiments. This method links the system’s quantum dynamics to electromagnetic fields, providing a bridge between quantum emitter dynamics and spatio-temporally resolved classical fields. We show that SDEs can properly account for the dynamics of open quantum systems and offer the potential for integration with Maxwell solvers^23,50,51^. Finally, we argue that deriving a stochastic Bloch vector from a non-symmetric diffusion matrix addresses a critical gap in the stochastic modeling of systems subject to non-trivial noise processes.

## Results

### SDEs for symmetric vs. non-symmetric diffusion matrices

In the following, we introduce the concept of SDEs for non-symmetric diffusion matrices (see the central Eqs. (7) and (10)). To better motivate this innovation, we start by briefly summarizing the standard path from a (quantum) master equation to an SDE relying on an ordinary symmetric diffusion matrix (see Eqs. (1) and (2)). After fixing the operator ordering, the master equation can be mapped to a phase-space quasi-probability distribution by replacing quantum operators with corresponding differential operators^33,52^. From there, one derives an FPE, whose main building blocks are the drift matrix **A**, responsible for the deterministic evolution, and the diffusion matrix **D**, describing the stochastic fluctuations^53^. In conventional treatments, **D** is typically symmetric and positive semidefinite, allowing a factorization^53,54^

1 $${{\bf{D}}}={{\bf{B}}}{{{\bf{B}}}}^{{{\rm{T}}}}\,,$$

that defines the noise coupling for the SDE via the noise matrix **B**. This factorization is not unique, a property that has been exploited in previous work^55,56^. The resulting linear Itô SDE with additive noise is^53^

2 $${\partial }_{t}{{\bf{s}}}(t)={{\bf{A}}}{{\bf{s}}}(t)+{{\bf{B}}}{{\boldsymbol{\xi }}}(t)\,,$$

where **s**(*t*) is the stochastic process that solves the SDE and ***ξ***(*t*) is a vector-valued Gaussian white noise process. However, the restriction to a symmetric **D** excludes crucial asymmetric noise correlations originating from non-commuting operators in the quantum realm. In the following, we show how to overcome this limitation by allowing a complex and non-symmetric **D**, which can be interpreted as a noise cross-covariance between two coupled stochastic processes.

**Fig. 1:**
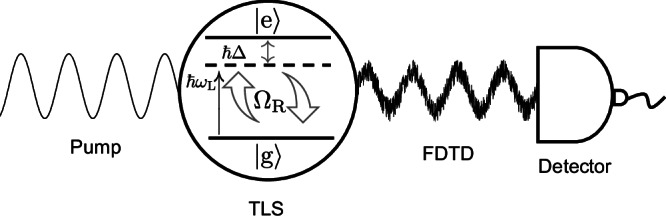
Schematic representation of the modeled system. From left to right: the two-level system (TLS), embedded in vacuum, is coherently driven by a classical field, inducing Rabi oscillations at the Rabi frequency *Ω*_R_ between the ground state $\left|g\right\rangle$ and excited state $\left|e\right\rangle$. The detuning energy *ℏΔ* quantifies how far the driving frequency is off-resonant from the natural transition frequency of the TLS, and *ℏω*_L_ denotes the laser photon energy. The stochastic trajectories of the TLS steady-state dynamics are coupled to a source current and are propagated using a finite-difference time-domain (FDTD) solver.

As a representative example, we consider a TLS driven coherently by a laser field (see Fig. 1). With the help of the pseudospin operators *σ*_±_ and *σ*_z_, it can be described by the Hamiltonian^57^

3 $$H=\frac{\hslash }{2}\left(\Delta {\sigma }_{{{\rm{z}}}}-{\Omega }_{{{\rm{R}}}}\left({\sigma }_{+}+{\sigma }_{-}\right)\right)\,,$$

where *Ω*_R_ denotes the Rabi frequency, and *Δ* = *ω*_0_ − *ω*_L_ is the detuning of the two-level transition frequency *ω*_0_ with respect to the laser frequency *ω*_L_. The TLS dynamics is governed by the standard Lindblad master equation in the Born–Markov and rotating wave approximation^20,57^. We choose the Lindblad jump operators *L*_1_ and *L*_2_ such that they take spontaneous emission and dephasing into account. Moving to Liouville space (see “Methods”), the Liouvillian superoperator in the Schrödinger picture^58^,

4 $${{\mathcal{L}}}=-{{\rm{i}}}{\hslash }^{-1}[[H,{{\mathbb{1}}}_{2}]]+{\sum }_{k=1}^{2}\left({L}_{k}\otimes {L}_{k}^{*}-\frac{1}{2}{[[{L}_{k}^{{\dagger} }{L}_{k},{{\mathbb{1}}}_{2}]]}_{+}\right)\,,$$

acts on the vectorized density matrix $|\rho (t)\rangle \rangle$. The steady state $|{\rho }_{{{\rm{s.s.}}}} \rangle \rangle$ is obtained from the zero-eigenvalue eigenmode of ${{\mathcal{L}}}$^57,59^,

5 $$|{\rho }_{{{\rm{s}}}.{{\rm{s}}}.}\rangle \rangle={{lim}}_{t \to {{\infty }}}{\sum }_{k}{{{\rm{e}}}}^{{\lambda }_{k}t}|{r}_{k}\rangle \rangle \langle \langle {l}_{k}|\rho (0)\rangle \rangle \,,$$

where *λ*_*k*_ are the complex eigenvalues of ${{\mathcal{L}}}$ with corresponding biorthonormal right and left eigenvectors $|{r}_{k}\rangle \rangle$ and $\langle \langle {l}_{k}|$. Here, the initial state $|\rho (0) \rangle \rangle$ is chosen to be the ground-state supervector $|{\sigma }_{{{\rm{gg}}}}\rangle \rangle$. Based on this, we define a second-order cumulant matrix ${{\bf{M}}}=({M}_{ij})\in {{\mathbb{C}}}^{3\times 3}$ for the pseudospin operators, which captures correlations in the steady state. Its entries are given by

6 $${M}_{ij}=\langle \langle {({\sigma }_{i}{\sigma }_{j})}^{{\dagger} }| {\rho }_{{{\rm{s.s.}}}}\rangle \rangle -\langle \langle {\sigma }_{i}^{{\dagger} }| {\rho }_{{{\rm{s.s.}}}}\rangle \rangle \langle \langle {\sigma }_{j}^{{\dagger} }| {\rho }_{{{\rm{s.s.}}}}\rangle \rangle \,,$$

where *i*, *j*∈{−, +, *z*}.

The system’s dynamics can be recast into two coupled linear SDEs, each driven by additive noise with a shared cross-covariance structure. We start by defining a non-symmetric diffusion matrix **D** as a linear combination of the second-order cumulant matrix **M** and the drift matrix **A,**

7 $$-{{\bf{D}}}={{\bf{A}}}{{\bf{M}}}+{{\bf{M}}}{{{\bf{A}}}}^{{{\rm{T}}}}\,,$$

which is reminiscent of an algebraic Lyapunov equation^53^. This observation fits well into recent work, which has revealed a deep connection between Lyapunov equations and open quantum systems^60^. Since in Eq. (7), **D** is a complex and non-symmetric matrix, it loses the meaning of a diffusion matrix as used in SDE theory. Instead, it turns out that **D** acts as the noise cross-covariance matrix. In order to factorize **D**, we use the singular value decomposition (SVD) instead of the Takagi factorization^56^, which enables us to decompose non-symmetric matrices by

8 $${{\bf{D}}}={{\bf{U}}}\,{{\boldsymbol{\Sigma }}}\,{{{\bf{V}}}}^{{\dagger} }\,,$$

where **U**, **V** are unitary matrices, and **Σ** is a diagonal matrix with non-negative real entries. We introduce the corresponding complex noise matrices

9 $${{{\bf{B}}}}_{1}={{\bf{U}}}\,\sqrt{{{\boldsymbol{\Sigma }}}}\,,\,{{{\bf{B}}}}_{2}={{{\bf{V}}}}^{*}\sqrt{{{\boldsymbol{\Sigma }}}}\,,$$

such that ${{{\bf{B}}}}_{1}{{{\bf{B}}}}_{2}^{{{\rm{T}}}}={{\bf{D}}}$. The noise matrices **B**_1_ and **B**_2_ together with the drift matrix **A** define a doubled system of linear SDEs with inhomogeneity **b** and additive noise (in Itô form, see “Methods” for details)

10 $${\partial }_{t}{{{\bf{s}}}}_{k}(t)={{\bf{A}}}{{{\bf{s}}}}_{k}(t)+{{\bf{b}}}+{{{\bf{B}}}}_{k}\,{{\boldsymbol{\xi }}}(t)\,,\,k\in \{1,2\}\,,$$

where for each *k* the vector-valued stochastic process ${{{\bf{s}}}}_{k}={({s}_{k,-}{s}_{k,+}{s}_{k,z})}^{{{\rm{T}}}}$, indexed in analogy to pseudospin operators, solves Eq. (10). Importantly, as we will see in the next section, this is not merely a labeling. Rather, the components of **s**_*k*_ fulfill an analog modeling role as their operator-valued counterparts. This representation allows us to interpret the dynamics as complex stochastic Bloch vector trajectories, whose ensemble behavior reproduces the correct steady-state correlations. To our knowledge, this use of an SVD factorization to construct two coupled SDEs with a prescribed cross-covariance has not been discussed in the quantum-optics literature. Most importantly, from the ensemble average of the stochastic Bloch vectors **s**_1_(*τ*), **s**_2_(0), we can compute two-time correlation functions, which would not be possible in a purely semiclassical approach. As we will demonstrate next, this allows us to simulate quantum features in optical experiments.

### Steady-state fluctuations and the Mollow spectrum

In the driven dissipative regime, the steady state of the TLS is subject to intrinsic quantum fluctuations. These manifest in time-resolved correlation functions and ultimately shape the emission spectrum^42^. In particular, the incoherent spectrum encodes deviations from the mean field due to quantum noise and is defined as the steady-state correlation of the fluctuation operator $\delta {\sigma }_{\pm }={\sigma }_{\pm }-{\langle {\sigma }_{\pm }\rangle }_{{{\rm{s.s.}}}}$^32^. Using the trajectories of our stochastic Bloch vectors (Eq. (10)), we construct the steady-state fluctuation correlation matrix by

11 $${{{\mathcal{C}}}}_{ij}^{{{\rm{sto}}}}(\tau )={\overline{({s}_{1,i}(\tau ){s}_{2,j}(0))}}_{{{\rm{s.s.}}}}-{\overline{({s}_{1,i})}}_{{{\rm{s.s.}}}}\,{\overline{({s}_{2,j})}}_{{{\rm{s.s.}}}}\,,$$

where *i*, *j*∈{−, +, *z*}. Taking the real part of the one-sided Fourier transform of ${{{\mathcal{C}}}}_{+-}^{{{\rm{sto}}}}(\tau )$,

12 $${S}_{{{\rm{inc}}}}^{{{\rm{sto}}}}(\omega )\propto {\mathrm{Re}} \left\{\int _{0}^{\infty }{{\rm{d}}}\tau \,{{{\rm{e}}}}^{-{{\rm{i}}}\omega \tau }{{{\mathcal{C}}}}_{+-}^{{{\rm{sto}}}}(\tau )\right\}\,,$$

provides the incoherent spectrum, namely the Mollow triplet, a hallmark of resonance fluorescence. Since the spectrum is evaluated in the rotating frame of the laser frequency *ω*_L_, the variable *ω* represents the detuning from the drive. The coherent part is given by

13 $${S}_{{{\rm{coh}}}}^{{{\rm{sto}}}}( \omega )=\pi \,{\overline{({s}_{1,+}{s}_{2,-})}}_{{{\rm{s.s.}}}}\delta ( \omega )\,,$$

such that the total emission spectrum reads as

14 $${S}^{{{\rm{sto}}}}(\omega )={S}_{{{\rm{inc}}}}^{{{\rm{sto}}}}+{S}_{{{\rm{coh}}}}^{{{\rm{sto}}}}\,.$$

**Fig. 2:**
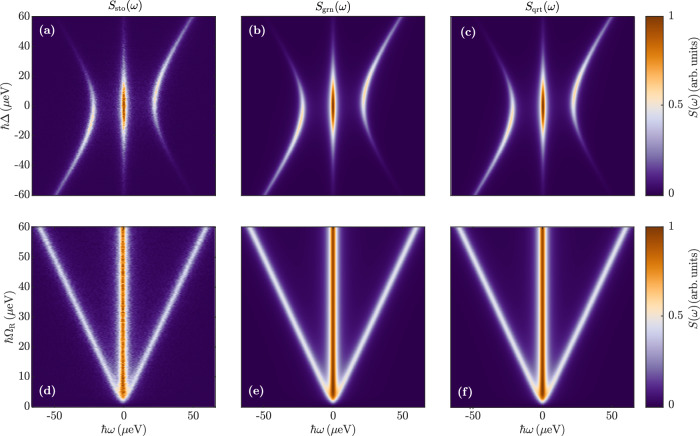
Maps of Mollow spectra obtained (from left to right) by stochastic approach (sto), Green’s propagator (grn), and quantum regression theorem (qrt). The presented data are normalized to their respective maxima. **a**–**c** show the Mollow spectrum *S*(*ω*) in dependence on the detuning energy *ℏΔ*, and **d**–**f** as a function of the Rabi energy *ℏΩ*_R_. Parameters used in the simulations are listed in Table 1.

Figure 2 shows the Mollow spectrum obtained from our stochastic approach, while systematically varying key parameters including the detuning (a) and driving strength (d). More precisely, Fig. 2a shows the total emission for a detuning ranging from −60 to 60 μeV, corresponding to values up to twice the Rabi energy (30 μeV) and therefore entering the large detuning regime. The Mollow triplet remains clearly resolved across the entire range, with sideband positions shifting and intensities evolving with detuning. In (d), we are on resonance (zero detuning) and increase the Rabi energy, leading to a clear separation of the Mollow triplet sidebands, while the central peak remains the dominant spectral feature. The stochastic nature of the simulation approach is revealed by the slight noise in (a) and (d). This noise is directly related to the number of realizations of the stochastic Bloch vector, in the following denoted as walkers.

**Fig. 3:**
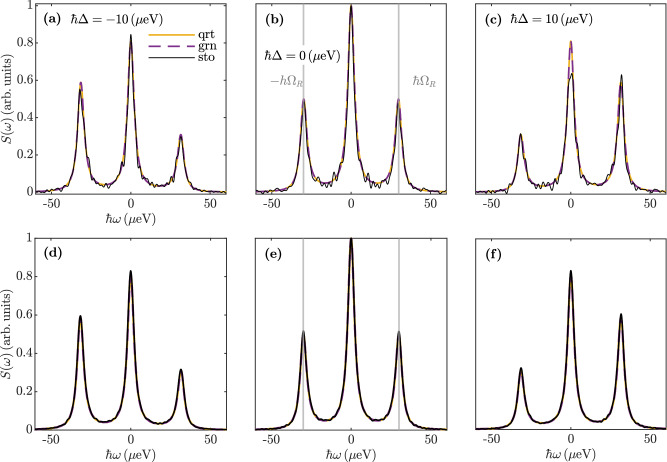
Lineplots of Mollow spectra obtained by the quantum regression theorem (yellow line, qrt), Green’s propagator (purple dashed line, grn), and stochastic approach (black line, sto). For convenience, the presented data are normalized to their respective maxima, which are equal up to numerical errors. Hence, this normalization does not introduce any distortions. The gray lines indicate the Rabi energy *ℏΩ*_R_. **a**–**f** show the Mollow spectrum *S*(*ω*) at a fixed Rabi energy with *ℏΩ*_R_ and varying detuning energy *ℏΔ*. The increase of the walkers *N* from **a**–**c** *N* = 4000, to **d**–**f** *N* = 100,000 shows the convergence of the stochastic approach. Simulation parameters: Table 1.

**Fig. 4:**
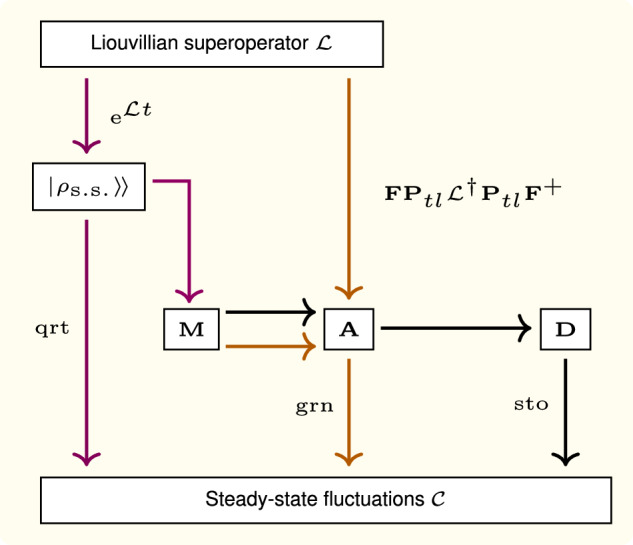
Outline of the derivation of the steady-state fluctuations. Purple line: the steady-state solution in Liouville space is obtained via time propagation using ${{{\rm{e}}}}^{{{\mathcal{L}}}t}$. From this, the second-order cumulant matrix **M** is derived. Additionally, by applying the quantum regression theorem, we directly obtain the steady-state fluctuations ${{\mathcal{C}}}$. Orange line: By taking the traceless part of the adjoint Liouvillian ${{{\mathcal{L}}}}^{{\dagger} }$ in the computational basis, the drift matrix **A** is obtained. Then, applying the matrix exponential e^**A***t*^ to the second-order cumulant matrix **M** yields the steady-state fluctuations ${{\mathcal{C}}}$. Black line: The noise cross-covariance matrix **D** is obtained as a linear combination of **M** and **A**. Factorizing **D** via singular value decomposition yields the noise matrices, which enable the construction of stochastic differential equations, leading to the steady-state fluctuations ${{\mathcal{C}}}$.

In order to quantify the influence of the number of walkers, we show in Fig. 3a–f that the stochastic approach (sto) converges to the established solution. This is done by comparing the Mollow spectra, where the underlying steady-state fluctuations are either obtained by use of the quantum regression theorem (qrt)^61^ or the Green’s propagator method (grn) (Eq. (16)), see Fig. 4. The steady-state fluctuations with the quantum regression theorem in Liouville space are given by^58^

15 $${{{\mathcal{C}}}}_{ij}^{{{\rm{qrt}}}}(\tau ) 	= \langle \langle {\sigma }_{i}{(\tau )}^{{\dagger} }| {\sigma }_{j}(0){\rho }_{{{\rm{s.s.}}}}\rangle \rangle -\langle \langle {\sigma }_{i}^{{\dagger} }| {\rho }_{{{\rm{s.s.}}}}\rangle \rangle \langle \langle {\sigma }_{j}^{{\dagger} }| {\rho }_{{{\rm{s.s.}}}}\rangle \rangle \\ 	={\sum}_{k=1}^{4}\,{{{\rm{e}}}}^{{\lambda }_{k}\tau }\,\langle \langle {\sigma }_{i}^{{\dagger} }| {r}_{k}\rangle \rangle \,\langle \langle {l}_{k}| {\sigma }_{j}{\rho }_{{{\rm{s.s.}}}}\rangle \rangle -\langle \langle {\sigma }_{i}^{{\dagger} }| {\rho }_{{{\rm{s.s.}}}}\rangle \rangle \langle \langle {\sigma }_{j}^{{\dagger} }| {\rho }_{{{\rm{s.s.}}}}\rangle \rangle .$$

For consistency, the steady-state fluctuations are also obtained directly from the drift matrix, the deterministic core of the SDE. We can project the adjoint Liouvillian ${{{\mathcal{L}}}}^{{\dagger} }$ onto the pseudospin basis to obtain a compact Bloch vector equation for the dynamics of single-time expectation values, see “Methods.” This yields a drift matrix **A** and an inhomogeneous term **b**, allowing us to recast the quantum regression theorem into a matrix propagation form

16 $$\begin{array}{rcl}{{{\mathcal{C}}}}_{ij}^{{{\rm{grn}}}}(\tau ) &=& \langle \langle {\sigma }_{i}{(\tau )}^{{\dagger} }| {\sigma }_{l}(0){\rho }_{{{\rm{s.s.}}}}\rangle \rangle -\langle \langle {\sigma }_{i}^{{\dagger} }| {\rho }_{{{\rm{s.s.}}}}\rangle \rangle \langle \langle {\sigma }_{l}^{{\dagger} }| {\rho }_{{{\rm{s.s.}}}}\rangle \rangle \\ &=& {\sum }_{k=1}^{4}\,{\left({{{\rm{e}}}}^{{{\bf{A}}}\tau }\right)}_{ik}{M}_{kj}\,.\end{array}$$

In this representation, the time evolution of fluctuations becomes a simple matrix exponential acting on the cumulant matrix **M**. Due to this structural similarity to linear response propagators, we refer to this as the Green’s (grn) propagator approach. The comparison of the detuned and power-dependent spectra in Fig. 2a, d *S*^sto^(*ω*), (b,e) *S*^grn^(*ω*), and (c,f) *S*^qrt^(*ω*), shows excellent agreement. Turning to Fig. 3, the comparison between panels (a)–(c), which use a relatively low number of walkers (*N* = 4 × 10^3^), shows that the overall behavior of the Mollow spectrum is captured quite well. Panels (d)–(f) use a larger number of walkers (*N* = 10^5^). This comparison demonstrates that the results obtained from our stochastic approach (sto) converge to the results from (grn) and (qrt).

We conclude this section by noting that in the stochastic approach, we do not explicitly fix an operator ordering during the derivation of the noise matrices **B**_*k*_ (Eq. (9)) and the associated SDEs (Eq. (10)). Instead, we construct the SDEs (Eq. (10)) directly from the noise cross-covariance structure **D** (Eq. (7)), without committing to a particular phase-space representation. This is in contrast to traditional methods, where the choice of ordering (e.g., normal, antinormal, symmetric) guides the mapping from quantum operators to classical stochastic variables. Nonetheless, the solutions to the resulting SDEs must be interpreted with an ordering consistent with the physical observables of the problem to correctly reproduce quantum correlations.

### Stochastic Bloch vector coupled to an FDTD solver

**Fig. 5:**
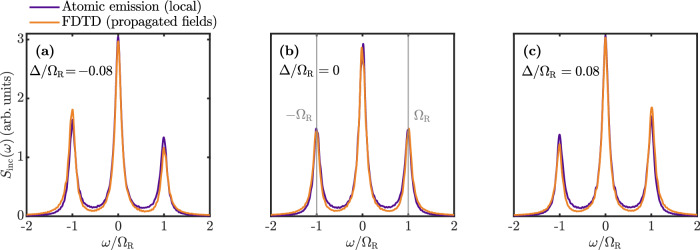
Comparison of the Mollow triplet under fixed Rabi driving, obtained from stochastic differential equations (at the atom, purple line) and from the propagated electric fields using the finite-difference time-domain (FDTD, orange line) method. The gray lines indicate the Rabi frequency *Ω*_R_. **a**–**c** show the Mollow triplet for slightly negative, zero, and positive detuning, respectively. Here, we express all frequencies in units of the Rabi frequency *Ω*_R_ and times in units of 1/*Ω*_R_, setting *Ω*_R_ = 1.

In the next step, we show that the trajectories of the stochastic Bloch vector can be coupled to a Maxwell solver. Here, we use the finite-difference time-domain (FDTD) method^12,62^, which allows us to directly propagate the electric and magnetic field in time. More precisely, we couple the stochastic Bloch vector components responsible for lowering and raising energy *s*_∓_(*t*) to an electromagnetic source via a spatial Gaussian centered around *μ* and broadened by *σ*,

17 $${{{\mathscr{J}}}}_{{{\rm{source}}}}^{\pm }(x,t)\,=\,\frac{1}{\sigma \sqrt{2\pi }}\,{{{\rm{e}}}}^{-\frac{{(x-\mu )}^{2}}{2{\sigma }^{2}}}\,{{{\rm{e}}}}^{\mp {{\rm{i}}}{\omega }_{{{\rm{c}}}}t}\,{s}_{\mp }(t)\,,$$

where *s*_−_(*t*): = *s*_1,−_(*t*), *s*_+_(*t*): = *s*_2,+_(*t*) and ${{{\rm{e}}}}^{\mp {{\rm{i}}}{\omega }_{{{\rm{c}}}}t}$ is the carrier-frequency phase factor. We choose *σ* ≪ 1 such that the Gaussian effectively approximates *δ*(*x*) while remaining numerically stable. For demonstration purposes, we use the source term (Eq. (17)) in a one-dimensional FDTD solver with first-order Mur boundary conditions. We propagate the positive and negative frequency (denoted ±) electric and magnetic field components by^63^

18 $$\begin{array}{rc}{\left.{{{\mathscr{E}}}}_{y}^{(\pm )}\right|}_{i}^{n+1} &={\left.{{{\mathscr{E}}}}_{y}^{(\pm )}\right|}_{i}^{n}-\frac{\Delta t}{\varepsilon \Delta x}\left({\left.{{{\mathscr{H}}}}_{z}^{(\pm )}\right|}_{i+\frac{1}{2}}^{n+\frac{1}{2}}-{\left.{{{\mathscr{H}}}}_{z}^{(\pm )}\right|}_{i-\frac{1}{2}}^{n+\frac{1}{2}}\right)\\ & -\frac{\Delta t}{\varepsilon }{\left.{{{\mathscr{J}}}}_{{{\rm{source}}}}^{(\pm )}\right|}_{i}^{n+\frac{1}{2}}\,,\end{array}$$

19 $${\left.{{{\mathscr{H}}}}_{z}^{(\pm )}\right|}_{i+\frac{1}{2}}^{n+\frac{1}{2}}={\left.{{{\mathscr{H}}}}_{z}^{(\pm )}\right|}_{i+\frac{1}{2}}^{n-\frac{1}{2}}-\frac{\Delta t}{\Delta x}\left({\left.{{{\mathscr{E}}}}_{y}^{(\pm )}\right|}_{i+1}^{n}-{\left.{{{\mathscr{E}}}}_{y}^{(\pm )}\right|}_{i}^{n}\right)\,,$$

where *ε* is the dielectric function, which we set to unity to model a vacuum environment, Δ*t* and Δ*x* denote the time step and spatial grid spacing, respectively, and the superscript *n* and subscript *i* refer to the temporal and spatial indices of the FDTD grid. In summary, the TLS radiates into the classical grid through the source current ${{{\mathscr{J}}}}_{{{\rm{source}}}}^{\pm }(x,t)$. This implementation enables the investigation of how vacuum-induced phenomena and incoherent emission manifest in nanophotonic structures. We now reformulate the incoherent part of the optical spectrum in terms of the positive- and negative-frequency electric field components (denoted by superscripts ±),

20 $$\begin{array}{rcl}{S}_{{{\rm{inc}}}}^{{{\rm{FDTD}}}} \propto {{\rm{Re}}}\left\{{\int }_{0}^{\infty }{{\rm{d}}}\tau \,{{{\rm{e}}}}^{-{{\rm{i}}}\omega \tau }\right. \left.\times \left({\overline{{{{\mathscr{E}}}}^{-}(\tau ){{{\mathscr{E}}}}^{+}(0)}}_{{{\rm{s.s.}}}}-{\overline{{{{\mathscr{E}}}}^{-}}}_{{{\rm{s.s.}}}}\,{\overline{{{{\mathscr{E}}}}^{+}}}_{{{\rm{s.s.}}}}\right)\right\}\,,\end{array}$$

where the overbar denotes an ensemble average over stochastic realizations of the electric field in the steady state. In practice, the two-time correlation is calculated by evaluating the positive- and negative-frequency field components at two times separated by *τ* within each realization and averaging the resulting products over all starting times and statistically independent realizations, see also “Methods” section. The incoherent contribution is obtained by subtracting the product of the corresponding steady-state mean fields. In Fig. 5, we plot the results of Eq. (20) together with those of Eq. (12) for a fixed Rabi driving and three different detuning scenarios. This provides a comparison between the Mollow triplet of the atomic emission (at the atom) and that obtained from the FDTD-propagated electric fields, both showing the expected spectral features.

The direct access to spatial and temporal electric fields provided by our approach involving the FDTD method is desirable since we deal with exactly those quantities that are accessible in experiments.

### Emission spectrum of an InGaAs quantum dot

For model validation, the simulation results are compared to experimental measurements on an InGaAs QD, where the negatively charged exciton serves as a TLS. For the measurement, the sample is cooled to 4.2 K and integrated into a resonance fluorescence setup to separate the laser signal from the QD emission by cross-polarized filtering. The emission is recorded with a high-resolution scanning Fabry–Perot interferometer. Using our stochastic approach, we successfully reproduce the power-dependent emission spectrum of a monochromatic resonantly driven QD for moderate Rabi energies. The model also captures the behavior of the QD emission under varying laser detuning.

**Fig. 6:**
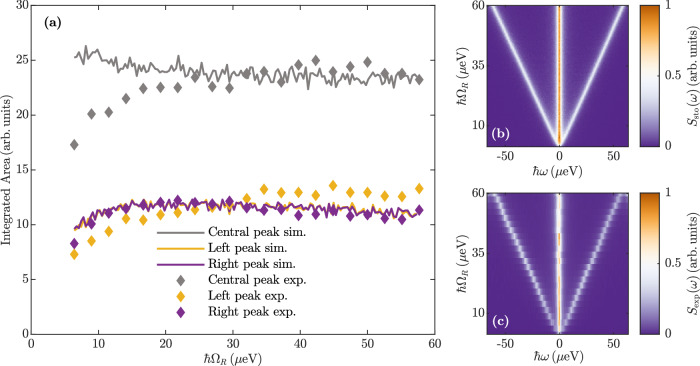
Power-dependent Mollow spectrum. Comparison of the stochastic approach with experimental data obtained from the InGaAs quantum dot. **a** Integrated peak areas of the Mollow spectrum, with solid lines corresponding to the stochastic approach and diamonds indicating the experimental results. **b** Power-dependent Mollow spectra (normalized) obtained by the stochastic approach. **c** Experimental power-dependent Mollow spectra (normalized). Simulation parameters: Table 1.

**Fig. 7:**
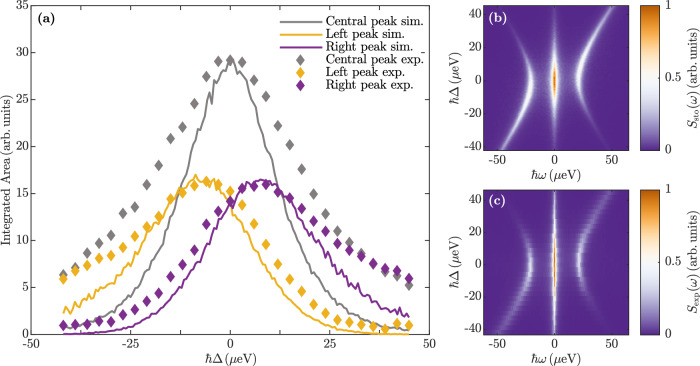
Detuning-dependent Mollow spectrum. Comparison of the stochastic approach with experimental data obtained from the InGaAs quantum dot. **a** Integrated peak areas of the Mollow spectrum, with solid lines corresponding to the stochastic approach and diamonds indicating the experimental results. **b** Detuning-dependent Mollow spectra (normalized) obtained by stochastic approach. **c** Experimental detuning-dependent Mollow spectra (normalized). Simulation parameters: Table 1.

We first investigate how the emission spectrum evolves with increasing laser power. A continuous wave laser tuned in resonance with the QD transition at the energy *E* = 1362.0315 meV is used, and the excitation power is increased from 1 nW to 2.21 μW in quadratically increasing steps. In Fig. 6c, we plot the experimental data against the Rabi energy $\hslash {\Omega }_{{{\rm{R}}}}=\sqrt{\hslash \,{\eta }_{{{\rm{R}}}}{P}_{{{\rm{exc}}}}}$ and the relative photon energy *ℏω*, where *η*_R_ is a dimensionless scaling factor. For comparison, we plot our stochastic approach in Fig. 6b, whereby the stochasticity is reflected in the slight noise in the data. To obtain a more quantitative comparison, we fit the spectra for each *ℏΩ*_R_ with Lorentzian line shapes and integrate the area under each peak (see Fig. 6a). The onset of Mollow triplet formation is observed for a Rabi energy *ℏΩ*_R_ > 6.4 μeV. For small Rabi energies, the peak areas extracted from experiment and simulation differ slightly, which we mainly attribute to difficulties in disentangling the contributions of the overlapping peaks. For characterizing the off-resonant behavior of the QD, we fix the driving strength at *ℏΩ*_R_ = 21 μeV, and detune the laser in equidistant steps from −42 to 45 μeV with respect to the QD *X*^−^ resonance. In Fig. 7b, c, we show the comparison of the experimental data and our stochastic approach. For each *ℏΔ*, we characterize the resulting spectrum by integrating the area under the fitted peaks, see Fig. 7a. The observed small differences between simulation and experiment in Figs. 6 and 7 can be attributed to experimental inaccuracies such as fluctuations and drifts in power and detuning, limited spectral resolution, laser induced heating, and a spectral dependence of the efficiency resulting from the cross-polarized suppression of the excitation laser. Here, we note that we also attempted to account for phonon effects in the Markovian regime^64–66^ by modifying the dephasing rate of the respective Lindblad jump operator, which did not lead to a significantly better agreement. To what extent the comparison of simulation and experiment can be improved using more advanced methods, such as the polaron master equation or variational polaron master equation^67,68^, lies beyond the scope of this work.

## Discussion

We demonstrate that allowing a non-symmetric diffusion matrix in the derivation of SDEs fills a critical gap in the modeling of non-trivial noise processes. As a quantum optical example showcasing the benefits of our approach, we accurately reproduce the power- and detuning-dependent emission spectrum of an InGaAs QD. Here, the resulting smooth transition from quantum to classical regimes guarantees compatibility with classical electromagnetic solvers.

The core of our formulation is a factorization of the non-symmetric diffusion matrix, which yields a doubled system of SDEs. In this work, we have employed SVDs for the factorization, but one could also use other schemes, such as matrix square roots, which might be beneficial for large matrices in order to speed up the simulations. The controlled distribution of noise contributions across these SDEs facilitates the encoding of correlated fluctuations that symmetric-diffusion models cannot represent. Conceptually, the non-symmetric diffusion matrix captures the consequences of operator non-commutativity in quantum mechanics. For the case of a TLS, which in our work is realized by an InGaAs QD, the system of SDEs may be viewed as a stochastic Bloch-vector description whose solutions can be injected into electromagnetic solvers via a source-current coupling. In doing so, it reproduces experimentally relevant spectral features, while retaining a possible path to device-scale modeling through the EM-coupling.

This is of particular interest because many Maxwell-Bloch solvers either neglect or approximate vacuum-induced fluctuations and therefore struggle to reproduce the incoherent part of the spectrum of a quantum emitter^21–26^. We are now able to naturally supply FDTD solvers with the missing quantum noise, which enables quantitative predictions of emission spectra in linear photonic environments without back action. While we here focus on the steady-state dynamics, the stochastic framework itself is not limited to steady-state conditions. Although our interface with FDTD does not yield a fully self-consistent simulation that includes the back-action on the quantum emitter, it can nevertheless model linear photonic environments that act as passive filters, such as optical cavities that transmit certain wavelengths and waveguides designed to pass specific modes.

Compared to prior stochastic approaches in quantum optics, most of which target ensembles of TLSs^39,69^, our approach addresses a single TLS without any approximations. Moreover, we anticipate no major obstacles standing in the way of an extension to multi-level systems using generalized Bloch vectors^22,70,71^.

Our derivation is currently established for two-time correlations. Many key observables in quantum optics fall into this category, and even second-order coherence can be handled when the required averages are recast in terms of two-time correlations^42^. Still, the extension to multi-time correlations and non-Gaussian noise processes is an attractive goal, since it enables the modeling of single photon emission^72,73^. In this work we have determined the noise matrices in Eq. (10) such that they lead to the proper equation of motion for the covariance matrix, see Eq. (46). We can speculate that along the same lines one could introduce higher-order noise contributions^74^ that have to be chosen such that they lead to the corresponding equations of motion for the higher-order correlation matrices, although further work is needed to work out the details.

In our current approach, we have introduced the relaxation and dephasing rates as parameters that have to be obtained from experiment or independent theoretical approaches. A particularly convenient one is the framework of macroscopic quantum electrodynamics^75^, which avoids the explicit quantization of electromagnetic fields by introducing material noise terms, and allows to derive optical decay rates in a first-principles manner. Quite generally, in the weak coupling regime, such rates could be easily obtained from supplementary FDTD simulations and introduced into our approach. In the strong-coupling regime, the mutual light–matter interaction has to be described explicitly^76^. Early work on Random Electrodynamics^77,78^ has shown that quantum effects in light–matter interactions can be mimicked through fluctuating classical fields, which has been used as the basis for the modern theory of the Casimir effect^78^. Although beyond the scope of this paper, we envision that within a related approach, the radiative decay of quantum emitters could be directly accounted for in FDTD through the consideration of the back-action of the emitted random fields on the quantum emitters. Future work should also address possible non-Markovian contributions in the dynamics of the quantum emitters, see ref. ^20^ for a detailed discussion.

From a computational perspective, our approach requires simulations for a sufficiently large number of stochastic noise realizations, which leads in general to significantly longer runtimes in comparison to more direct approaches. However, the major achievement of this work is the demonstration that genuine quantum effects can be modeled with classical FDTD solvers, despite the disbelief and reservations of the community. For example, a possible caveat is that the quantum-classical correspondence is believed to fail for certain limiting cases, such as the single two-level atom^14^. Considering that the approach holds potential for further improvements, such as the self-consistent treatment of light–matter interactions discussed above, and the fact that classical FDTD solvers can be employed without any major modification, we think that the overhead in computer time is acceptable, in particular in view of the fast performance of FDTD simulations and the possibility of parallelization. However, one can also envision scenarios where our approach in its present form can already outperform more traditional approaches. A new generation of spectroscopy and microscopy techniques relies on photon correlation measurements for multiple emitters, harnessing all possible kinds of spatial, temporal, and spectral correlations^79^. In a traditional approach, one first has to characterize the field propagators for all possible source and field positions as well as delay times, before convoluting them with the sources. In contrast, in our approach, the quantum properties of the sources are directly imprinted on the fields, and photon correlations can be computed from the simulated fields at different positions and times in a massively parallel fashion without explicitly establishing a link between sources and fields. In such setups, our approach appears to be superior to the traditional approaches, both regarding conceptual simplicity and computational speed.

Finally, despite the fact that our approach removes operator ordering on the quantum side, it relocates the choice to the classical post-processing stage. One must adopt a consistent ordering when mapping stochastic variables to observables. Notably, recent research has focused on filtering out the coherent and incoherent components of emission spectra, a task that our framework could support^80–82^.

In conclusion, our approach offers a practical route to quantitatively link quantum noise and classical fields, while also providing a general methodology for tailoring correlated noise in stochastic models beyond quantum optics.

## Methods

We adopt the following notation: (⋅)^T^ denotes the transpose, (⋅)^*^ denotes the complex conjugate, ${(\cdot )}^{{\dagger} }={({(\cdot )}^{*})}^{{{\rm{T}}}}$ denotes the Hermitian adjoint, 〈⋅〉 denotes the expectation value as used in quantum theory, and $\overline{(\cdot )}$ denotes the sample mean. Unless stated otherwise, the parameters used for each figure in this manuscript are listed in Table 1.

**Table 1:** Simulation parameters

Description	Symbol	Fig. 2a–c	Fig. 2d–f	Fig. 3	Fig. 6	Fig. 7
Rabi energy	*ℏΩ*_R_ (μeV)	30	[0, 60]	30	[0, 60]	21
detuning energy	*ℏΔ* (μeV)	[−60, 60]	0	[−10, 10]	0	[−42, 45]
relaxation time	*T*_1_ (ps)	263	263	263	296	296
dephasing time	*T*_2_ (ps)	527	527	527	987	592
number of walkers	*N*	4 × 10^3^	4 × 10^3^	4 × 10^3^, 10^5^	4 × 10^3^	15 × 10^4^

### Pseudospin operators

The following convention is used for the energy eigenstates of the TLS (ground state $\left|g\right\rangle$ and excited state $\left|e\right\rangle$):

21 $$\left|g\right\rangle=\left(\begin{array}{r}0\\ 1\\ \end{array}\right)\,,\,\left|e\right\rangle=\left(\begin{array}{r}1\\ 0\\ \end{array}\right).$$

We write the pseudospin operators as outer products regarding our chosen basis ${\sigma }_{ij}=|i\rangle \langle \;j|$ with *i*, *j*∈{*e*, *g*}, which are elements of the space ${{\mathcal{B}}}({{\mathbb{C}}}^{2})$ of bounded linear operators on the Hilbert space ${{\mathbb{C}}}^{2}$. However, we index the pseudospin operators in a way that reflects their physical action, using −, +, *z* instead of *e*, *g*, see Table 2.

**Table 2:** Matrix representation of pseudospin operators

Operator	Matrix representation
Lowering *σ*_−_	$${\sigma }_{-}:=|g\rangle \langle e|=\left(\begin{array}{rc}0 & 0\\ 1 & 0\\ \end{array}\right)$$
Raising *σ*_+_	$${\sigma }_{+}:=|e\rangle \langle g|=\left(\begin{array}{rc}0 & 1\\ 0 & 0\\ \end{array}\right)$$
Population difference *σ*_*z*_	$${\sigma }_{z}:=|e\rangle \langle e|-|g\rangle \langle g|=\left(\begin{array}{rc}1 & 0\\ 0 & -1\\ \end{array}\right)$$

These operators satisfy the commutation relations:

$$[{\sigma }_{z},{\sigma }_{\pm }]=\pm 2{\sigma }_{\pm }\,,\,\,[{\sigma }_{+},{\sigma }_{-}]={\sigma }_{z}\,.$$

### Liouville space

The formulation in Liouville space resembles Dirac’s bracket notation, and is primarily chosen for its readability and structural clarity. We work with the Hilbert space ${{\mathcal{H}}}={{\mathbb{C}}}^{2}$ for a TLS, on which operators act as 2 × 2 complex matrices. The Liouville space ${{\mathbb{L}}}_{2}$ is defined as the vector space isomorphic to ${{\mathbb{C}}}^{4}$, obtained by vectorizing operators on ${{\mathcal{H}}}$. We adopt the following convention for vectorization of operators $|a\rangle \langle b|\in {{\mathcal{B}}}({{\mathbb{C}}}^{2})$ to Liouville space ${{\mathbb{L}}}_{2}$

22 $${{\rm{vec}}}\left(|a\rangle \langle b|\right)=|a\rangle \otimes {|b\rangle }^{*}=|a,b \rangle \rangle \,,$$

where ⊗ denotes the Kronecker product, and $\left|\cdot \right\rangle \rangle$ indicates a superket, i.e., a vectorized operator in Liouville space. The corresponding superbra is defined as

23 $$\langle \langle a,b|:=\langle a|\otimes {\langle b|}^{*}\,,$$

so that the Hilbert-Schmidt inner product between operators *A* and *B* satisfies^58^

24 $$\langle \langle A| B\rangle \rangle :={{\rm{Tr}}}({A}^{{\dagger} }B)={{\rm{vec}}}{(A)}^{{\dagger} }{{\rm{vec}}}(B)\,.$$

Under this convention, pseudospin operators, originally 2 × 2 matrices on the Hilbert space ${{\mathbb{C}}}^{2}$, are mapped to 4 × 1 vectors in Liouville space. Their vectorized forms are shown in Table 3.

**Table 3:** Vectorization of pseudospin operators in Liouville space

Operator	Superket
Lowering *σ*_−_	$$|{\sigma }_{-}\rangle \rangle={{\rm{vec}}}(|g\rangle \langle e|)=\left(\begin{array}{r}0\\ 0\\ 1\\ 0\end{array}\right)$$
Raising *σ*_+_	$$|{\sigma }_{+}\rangle \rangle={{\rm{vec}}}(\left|e\right\rangle \left\langle g\right|)=\left(\begin{array}{r}0\\ 1\\ 0\\ 0\end{array}\right)$$
Population difference *σ*_*z*_	$$|{\sigma }_{z}\rangle \rangle={{\rm{vec}}}(\left|e\right\rangle \left\langle e\right|-\left|g\right\rangle \left\langle g\right|)=\left(\begin{array}{r}1\\ 0\\ 0\\ -1\end{array}\right)$$
Ground state *σ*_gg_	$$|{\sigma }_{{{\rm{gg}}}}\rangle \rangle={{\rm{vec}}}(\left|g\right\rangle \langle g|)=\left(\begin{array}{r}0\\ 0\\ 0\\ 1\end{array}\right)$$

The vectorization map ${{\rm{vec}}}:{{\mathcal{B}}}({{\mathbb{C}}}^{2})\to {{\mathbb{L}}}_{2}$ is linear and preserves the Hilbert-Schmidt inner product, making it a useful tool to translate operator equations into vector equations. Additionally, we define superoperators as elements of the space ${{\mathcal{B}}}({{\mathbb{L}}}_{2})$, the set of bounded linear operators acting on the Liouville space ${{\mathbb{L}}}_{2}$. These superoperators are represented by 4 × 4 complex matrices. For example, the Liouvillian superoperator ${{\mathcal{L}}}\in {{\mathbb{C}}}^{4\times 4}$ governs the dynamics of density operators in Liouville space. The super(anti)commutator maps $[[\cdot,\cdot ]]:{{\mathcal{B}}}({{\mathbb{C}}}^{2})\times {{\mathcal{B}}}({{\mathbb{C}}}^{2})\to {{\mathcal{B}}}({{\mathbb{L}}}_{2})$, so for $X,\,Y\in {{\mathcal{B}}}({{\mathbb{C}}}^{2})$, the supercommutator is defined as^83^

25 $$[[X,Y]]=X\otimes {Y}^{{{\rm{T}}}}-Y\otimes {X}^{{{\rm{T}}}}\,,$$

and the superanticommutator is given by

26 $${[[X,Y]]}_{+}=X\otimes {Y}^{{{\rm{T}}}}+Y\otimes {X}^{{{\rm{T}}}}\,.$$

To obtain expectation values and second-order moments for the vectorized pseudospin operators, we can adopt the formalism outlined above, yielding

27 $${\langle {\sigma }_{i}\rangle }_{{{\rm{s.s.}}}} = {{\rm{Tr}}}({\sigma }_{i}{\rho }_{{{\rm{s.s.}}}}) ={{\rm{Tr}}}\left({({\sigma }_{i}^{{\dagger} })}^{{\dagger} }{\rho }_{{{\rm{s.s.}}}}\right)=\langle \langle {\sigma }_{i}^{{\dagger} }| {\rho }_{{{\rm{s.s.}}}}\rangle \rangle $$

and

28 $${\langle {\sigma }_{i}{\sigma }_{j}\rangle }_{{{\rm{s.s.}}}} = {{\rm{Tr}}}({\sigma }_{i}{\sigma }_{j}{\rho }_{{{\rm{s.s.}}}}) = {{\rm{Tr}}}\left({\left({({\sigma }_{i}{\sigma }_{j})}^{{\dagger} }\right)}^{{\dagger} }{\rho }_{{{\rm{s.s.}}}}\right)=\langle \langle {({\sigma }_{i}{\sigma }_{j})}^{{\dagger} }| {\rho }_{{{\rm{s.s.}}}}\rangle \rangle.$$

### Second-order cumulant matrix

The Lindblad master equation in its typical form reads^16,20^

29 $${\partial }_{t}\rho=-{{\rm{i}}}{\hslash }^{-1}[H,\rho ]+{\sum }_{k}\left({L}_{k}\rho {L}_{k}^{{\dagger} }-\frac{1}{2}\{{L}_{k}^{{\dagger} }{L}_{k}\rho \}\right)\,,$$

where *L*_*k*_ are the Lindblad jump operators. Here, we apply the Lindblad master equation approach in a two-dimensional Liouville space ${{\mathbb{L}}}_{2}$. The Liouvillian superoperator ${{\mathcal{L}}}$ for a TLS in the Schrödinger picture can be expressed through

30 $${{\mathcal{L}}}=-{{\rm{i}}}{\hslash }^{-1}[[H,{{\mathbb{1}}}_{2}]]+\sum\limits_{k=1}^{2}\left({L}_{k}\otimes {L}_{k}^{*}-\frac{1}{2}{[[{L}_{k}^{{\dagger} }{L}_{k},{{\mathbb{1}}}_{2}]]}_{+}\right)\,,$$

where ${{\mathbb{1}}}_{2}$ is the two-dimensional identity operator. In our setting, the Lindblad jump operators are defined as ${L}_{1}=\sqrt{{\Gamma }_{1}}{\sigma }_{-}$ and ${L}_{2}=\sqrt{{\Gamma }_{2}}({{\mathbb{1}}}_{2}+{\sigma }_{z})/2$, where *Γ*_1_ = 1/*T*_1_ and *Γ*_2_ = 2/*T*_2_. Here, *T*_1_ denotes the relaxation time, while *T*_2_ is the phenomenological dephasing time that accounts for processes such as phonon-induced dephasing^57^. Plugging Eq. (3) and the Lindblad jump operators in Eq. (30) yields

31 $${{\mathcal{L}}}=\left(\begin{array}{llll}-{\Gamma }_{1} & \frac{-{{\rm{i}}}{\Omega }_{{{\rm{R}}}}}{2} & \frac{{{\rm{i}}}{\Omega }_{{{\rm{R}}}}}{2} & 0\\ \frac{-{{\rm{i}}}{\Omega }_{{{\rm{R}}}}}{2} & -{{\rm{i}}}\Delta -\frac{1}{2}\left({\Gamma }_{1}+{\Gamma }_{2}\right) & 0 & \frac{{{\rm{i}}}{\Omega }_{{{\rm{R}}}}}{2}\\ \frac{{{\rm{i}}}{\Omega }_{{{\rm{R}}}}}{2} & 0 & {{\rm{i}}}\Delta -\frac{1}{2}\left({\Gamma }_{1}+{\Gamma }_{2}\right) & \frac{-{{\rm{i}}}{\Omega }_{{{\rm{R}}}}}{2}\\ {\Gamma }_{1} & \frac{{{\rm{i}}}{\Omega }_{{{\rm{R}}}}}{2} & \frac{-{{\rm{i}}}{\Omega }_{{{\rm{R}}}}}{2} & 0\end{array}\right).$$

The time-independent Liouvillian superoperator (Eq. (30)) is known to be the generator of a quantum Markovian evolution, i.e., it generates a completely positive, trace-preserving semigroup $\{{{{\rm{e}}}}^{t{{\mathcal{L}}}}| t\ge 0\}$ under the Born–Markov and secular approximations^84,85^. The dynamics for the vectorized density matrix in Liouville space is then governed by

32 $$|\rho (t)\rangle \rangle={{{\rm{e}}}}^{{{\mathcal{L}}}t}|\rho (0)\rangle \rangle \,.$$

To compute this explicitly, we diagonalize ${{\mathcal{L}}}$ and expand the solution in terms of its biorthonormal eigenbasis $\{|{r}_{k}\rangle \rangle,\langle \langle {l}_{k}|\}$, yielding

33 $$|\rho (t)\rangle \rangle={\sum }_{k=1}^{4}{{{\rm{e}}}}^{{\lambda }_{k}t}|{r}_{k}\rangle \rangle \langle \langle {l}_{k}| \rho (0)\rangle \rangle \,,$$

where *λ*_*k*_ are the complex eigenvalues of ${{\mathcal{L}}}$, with corresponding right eigenvectors $|{r}_{k}\rangle \rangle$ and left eigenvectors $\langle \langle {l}_{k}|$.

We obtain the steady-state solutions in the long-time limit $|{\rho }_{{{\rm{s.s.}}}}\rangle \rangle :={lim}_{t\to \infty }|\rho (t)\rangle \rangle$ via

34 $$|{\rho }_{{{\rm{s.s.}}}}\rangle \rangle=\sum\limits_{{k}_{\operatorname{Re}\{\lambda_k\}=0}}|{r}_{k}\rangle \rangle \langle \langle {l}_{k}| {\sigma }_{{{\rm{gg}}}}\rangle \rangle \,,$$

where the ground-state supervector $|{\sigma }_{{{\rm{gg}}}}\rangle \rangle$, see Table 3, is used as an initial state. Here we have used those terms with ${{\rm{Re}}}\{{\lambda }_{k}\} < 0$ decay as *t* → *∞* and therefore do not contribute to the steady state.

We define the steady-state second-order cumulant matrix for the pseudospin operators as

35 $${{\bf{M}}}={({M}_{ij})}_{i,j\in \{-,+,z\}}\in {{\mathbb{C}}}^{3\times 3}\,,$$

with entries

36 $${M}_{ij}=\langle \langle {({\sigma }_{i}{\sigma }_{j})}^{{\dagger} }| {\rho }_{{{\rm{s.s.}}}}\rangle \rangle -\langle \langle {\sigma }_{i}^{{\dagger} }| {\rho }_{{{\rm{s.s.}}}}\rangle \rangle \langle \langle {\sigma }_{j}^{{\dagger} }| {\rho }_{{{\rm{s.s.}}}}\rangle \rangle \,.$$

In the Heisenberg picture, operators evolve under the adjoint Liouvillian as ${\sigma }_{i}(\tau )={{{\rm{e}}}}^{{{{\mathcal{L}}}}^{{\dagger} }\tau}{\sigma }_{i}$. Using this, the quantum regression theorem in Liouville space can therefore be written as

37 $$\begin{array}{rcl}{\langle {\sigma }_{i}(\tau ){\sigma }_{j}(0)\rangle }_{{{\rm{s.s.}}}} &=& {{\rm{Tr}}}({\sigma }_{i}(\tau ){\sigma }_{j}(0){\rho }_{{{\rm{s.s.}}}})\\ &=& \langle \langle {\sigma }_{i}{(\tau )}^{{\dagger} }| {\sigma }_{j}(0){\rho }_{{{\rm{s.s.}}}}\rangle \rangle \\ &=& \left\langle \left\langle {\sigma }_{i}^{{\dagger} }\right|{{{\rm{e}}}}^{{{\mathcal{L}}}\tau }\left|{\sigma }_{j}(0){\rho }_{{{\rm{s.s.}}}}\right\rangle \right\rangle \\ &=& {\sum }_{k=1}^{4}{{{\rm{e}}}}^{{\lambda }_{k}\tau }\langle \langle {\sigma }_{i}^{{\dagger} }| {r}_{k}\rangle \rangle \langle \langle {l}_{k}| {\sigma }_{j}(0){\rho }_{{{\rm{s.s.}}}}\rangle \rangle \,.\end{array}$$

### Projection of ${{{\mathcal{L}}}}^{{\dagger} }$

We can project the adjoint Liouvillian ${{{\mathcal{L}}}}^{{\dagger} }$ by suitable matrix transformations into the computational basis, which yields the drift matrix **A** and inhomogeneity term **b**. The drift matrix **A** is known to be the deterministic time evolution matrix for Heisenberg-Langevin equations^33^. For a TLS, it is given by the traceless part of ${{{\mathcal{L}}}}^{{\dagger} }$ in the computational basis. We define

38 $${{{\bf{P}}}}_{{{\rm{tl}}}}={{\mathbb{1}}}_{4}-\frac{1}{2}|{{\mathbb{1}}}_{2}\rangle \rangle \langle \langle {{\mathbb{1}}}_{2}|$$

as the projection onto the traceless subspace. Furthermore, we define the matrix

39 $${{\bf{F}}}=|0\rangle \langle \langle {\sigma }_{-}|+|1\rangle \langle \langle {\sigma }_{+}|+|2\rangle \langle \langle {\sigma }_{z}|$$

that maps the basis of the traceless subspace $\{|{\sigma }_{-}\rangle \rangle,|{\sigma }_{+}\rangle \rangle,|{\sigma }_{z}\rangle \rangle \}$ into the three-dimensional computational basis $\{|0\rangle={(1,0,0)}^{{{\rm{T}}}},|1\rangle={(0,1,0)}^{{{\rm{T}}}},|2\rangle={(0,0,1)}^{{{\rm{T}}}}\}$. Thus, **F** is a 3 × 4 rectangular matrix. Denoting the Moore-Penrose inverse^86^ of **F** by **F**^+^, we have

40 $${{\bf{A}}}={{\bf{F}}}{{{\bf{P}}}}_{{{\rm{tl}}}}{{{\mathcal{L}}}}^{{\dagger} }{{{\bf{P}}}}_{{{\rm{tl}}}}{{{\bf{F}}}}^{+}\,,$$

which yields

41 $${{\bf{A}}}=\left(\begin{array}{rcl}-{{\rm{i}}}\Delta -\frac{{\Gamma }_{1}+{\Gamma }_{2}}{2} & 0 & -\frac{{{\rm{i}}}{\Omega }_{{{\rm{R}}}}}{2}\\ 0 & {{\rm{i}}}\Delta -\frac{{\Gamma }_{1}+{\Gamma }_{2}}{2} & \frac{{{\rm{i}}}{\Omega }_{{{\rm{R}}}}}{2}\\ -{{\rm{i}}}{\Omega }_{{{\rm{R}}}} & {{\rm{i}}}{\Omega }_{{{\rm{R}}}} & -{\Gamma }_{1}\end{array}\right)\,.$$

The inhomogeneity **b** is obtained by

42 $${{\bf{b}}}=\langle \langle {{\mathbb{1}}}_{2}|{{{\bf{P}}}}_{{{\rm{t}}}}{{{\mathcal{L}}}}^{{\dagger} }{{{\bf{P}}}}_{{{\rm{tl}}}}{{{\bf{F}}}}^{+}\,,$$

where ${{{\bf{P}}}}_{{{\rm{t}}}}={{\mathbb{1}}}_{4}-{{{\bf{P}}}}_{{{\rm{tl}}}}$ projects on the trace subspace, yielding

43 $${{\bf{b}}}=\left(\begin{array}{r}0\\ 0\\ -\frac{1}{{T}_{1}}\end{array}\right)\,.$$

With **A** and **b** at hand, one can construct a Bloch vector equation for the expectation values of the pseudospin operators in the Heisenberg picture,

44 $${\partial }_{t}{{\bf{u}}}={{\bf{A}}}{{\bf{u}}}+{{\bf{b}}}\,,$$

where $${{\bf{u}}}={(\langle {\sigma }_{-}(t)\rangle,\langle {\sigma }_{+}(t)\rangle,\langle {\sigma }_{z}(t)\rangle )}^{{{\rm{T}}}}$$.

We can also apply the quantum regression theorem using the drift matrix **A**. The entries of the steady-state fluctuations are given by

45 $${{{\mathcal{C}}}}_{ij}^{{{\rm{grn}}}}(\tau )={\sum }_{k=1}^{4}\,{\left({{{\rm{e}}}}^{{{\bf{A}}}\tau }\right)}_{ik}{M}_{kj}\,,$$

where the Green’s propagator method is primarily used as a consistency check for benchmarking our stochastic approach since it directly involves the drift matrix **A**, i.e., the deterministic part of the SDE, rather than the full Liouvillian.

### Stochastic approach

We follow the standard theory of linear SDEs, matrix analysis, and Gaussian processes^53,87–89^. The main modification is a non-symmetric diffusion matrix. The second-order cumulants do not depend on any particular operator ordering. The non-commutativity of the operators involved is responsible for the non-symmetric matrix **D**. The equation of motion for the covariance matrix **C** is given by^14^

46 $${\partial }_{t}{{\bf{C}}}={{\bf{A}}}{{\bf{C}}}+{{\bf{C}}}{{{\bf{A}}}}^{{{\rm{T}}}}+{{\bf{D}}}\,,$$

where **A** and **D** are the drift and diffusion matrices, respectively. In the steady state, this gives the (time-continuous) Lyapunov equation

47 $$-{{\bf{D}}}={{\bf{A}}}{{\bf{C}}}+{{\bf{C}}}{{{\bf{A}}}}^{{{\rm{T}}}}\,.$$

If **D** ≽ 0 and **A** is Hurwitz (i.e., all eigenvalues of **A** have strictly negative real parts), the unique steady-state solution **C** is positive semidefinite, ensuring that all variances (diagonal elements) are non-negative^53^. Rather than solving Eq. (47) for the covariance **C**, we express the diffusion matrix **D** as a linear combination of the drift matrix **A** and the second-order cumulant **M**

48 $$-{{\bf{D}}}={{\bf{A}}}{{\bf{M}}}+{{\bf{M}}}{{{\bf{A}}}}^{{{\rm{T}}}}\,.$$

This yields a complex, non-symmetric matrix **D**, which no longer has the standard interpretation of a diffusion matrix in SDE theory. Rather, **D** is interpreted as the noise cross-covariance matrix. To factorize **D**, we use the SVD Eq. (8), and construct the corresponding complex noise matrices according to Eq. (9). The noise matrices **B**_1_ and **B**_2_, together with the drift matrix **A**, define two linear SDEs with additive noise in Itô form

49 $$\left\{\begin{array}{l}{{\rm{d}}}{{{\bf{X}}}}_{k,t}=\left({{\bf{A}}}{{{\bf{X}}}}_{k,t}+{{\bf{b}}}\right){{\rm{d}}}t+{{{\bf{B}}}}_{k}\,{{\rm{d}}}{{\bf{W}}}(t),\\ {{{\bf{X}}}}_{k,0}={{{\bf{x}}}}_{k,0},\end{array}\right. \,\, \, k\in \{1,2\}\,,$$

where **X**_*k*,*t*_ is a stochastic process representing the system state, **b** is a constant inhomogeneous term, **W**(*t*) is a vector of independent real-valued Wiener processes, and **x**_*k*,0_ denotes the (possibly random) initial condition^53^.

Since *T*_1_, *T*_2_, *Ω*_R_ > 0, the drift matrix **A** is invertible and Hurwitz, which ensures that the dynamics relax to a stationary state^53,90^. Eq. (49) admits the analytical solution

50 $${{{\bf{X}}}}_{k,t}={{{\rm{e}}}}^{{{\bf{A}}}t}\left({{{\bf{x}}}}_{k,0}+{{{\bf{A}}}}^{-1}{{\bf{b}}}\right)-{{{\bf{A}}}}^{-1}{{\bf{b}}}+\int _{0}^{t}{{{\rm{e}}}}^{{{\bf{A}}}(t-\tau )}{{{\bf{B}}}}_{k}\,{{\rm{d}}}{{\bf{W}}}(\tau ).$$

At long times, the influence of the initial condition decays and the process converges to a stationary complex Gaussian process with mean^91^

51 $${\mathbb{E}}[{{{\bf{X}}}}_{k,\infty }]=-\,{{{\bf{A}}}}^{-1}{{\bf{b}}}$$

and covariance

52 $${{{\bf{C}}}}_{\infty }=\int _{0}^{\infty }{{{\rm{e}}}}^{{{\bf{A}}}s}\,{{{\bf{B}}}}_{k}{{{\bf{B}}}}_{k}^{{\dagger} }\,{{{\rm{e}}}}^{{{{\bf{A}}}}^{{\dagger} }s}\,{{\rm{d}}}s\,,$$

which is the unique Hermitian solution of the Lyapunov equation

53 $${{\bf{A}}}\,{{{\bf{C}}}}_{\infty }+{{{\bf{C}}}}_{\infty }{{{\bf{A}}}}^{{\dagger} }+{{{\bf{B}}}}_{k}{{{\bf{B}}}}_{k}^{{\dagger} }={{\bf{0}}}\,.$$

Thus, the stationary dynamics are fully described by a complex Gaussian distribution with mean  − **A**^−1^**b** and covariance **C**_*∞*_.

In conclusion, we note that in SDE theory, the diffusion matrix is often defined as **D** = **BB**^T^, such that the Itô isometry yields ${\mathbb{E}}[{{\rm{d}}}{{{\bf{X}}}}_{t}\,{{\rm{d}}}{{{\bf{X}}}}_{t}^{{{\rm{T}}}}]={{\bf{D}}}\,{{\rm{d}}}t$^92^. However, in our setting, this relation does not hold individually for each process. Instead, we find that the noise cross-covariance between the two processes is given by

54 $${\mathbb{E}}[{{\rm{d}}}{{{\bf{X}}}}_{t,1}\,{{\rm{d}}}{{{\bf{X}}}}_{t,2}^{{{\rm{T}}}}]={{{\bf{B}}}}_{1}{{{\bf{B}}}}_{2}^{{{\rm{T}}}}\,{{\rm{d}}}t={{\bf{D}}}\,{{\rm{d}}}t\,,$$

which defines a shared diffusion structure through the matrix **D**.

### Numerical simulation methods

For the numerical solution scheme, we now adapt a notation more common in physics and introduce the index *n*, which denotes the number of walkers, i.e., the number of stochastic trajectories

55 $${\partial }_{t}{{{\bf{s}}}}_{k}^{(n)}(t)={{\bf{A}}}{{{\bf{s}}}}_{k}^{(n)}(t)+{{\bf{b}}}+{{{\bf{B}}}}_{k}{{{\boldsymbol{\xi }}}}^{(n)}(t)\,,\,\begin{array}{l}k=1,2\,,\\ n=1,\ldots,N\,,\end{array}$$

where ${{{\bf{s}}}}_{k}={({s}_{k,-}{s}_{k,+}{s}_{k ,z})}^{{{\rm{T}}}}$, and the formal derivative ***ξ***(*t*): = d**W**(*t*)/*dt* is a real-valued vector white noise process in the sense of generalized functions ${\mathbb{E}}[{{\boldsymbol{\xi }}}(t)\,{{\boldsymbol{\xi }}}{(t{\prime} )}^{{{\rm{T}}}}]=\delta (t-t{\prime} )\,{{\mathbb{1}}}_{3}$ and each component of ***ξ***(*t*) has zero mean^93^. Comparing Eq. (55) with Eq. (44) suggests interpreting Eq. (55) as a stochastic Bloch vector. We solve Eq. (10) numerically by an exponential Euler-Maruyama method

56 $${{{\bf{s}}}}_{k}^{(n)}(m+1)={{{\rm{e}}}}^{{{\bf{A}}}\Delta t}{{{\bf{s}}}}_{k}^{(n)}(m)+{{\bf{b}}}\Delta t+\sqrt{\Delta t}\,{{{\bf{B}}}}_{k}\,{{{\boldsymbol{\xi }}}}^{(n)}(m)\,,$$

with ${{\boldsymbol{\xi }}}(m) \sim {{\mathcal{N}}}({{\bf{0}}},{{\mathbb{1}}}_{3})\,,\,{{\rm{i.i.d.\; over}}}\,m$ and *n* = 1,..., *N*. The deterministic part is treated exactly with the matrix exponential, and the stochastic part is integrated using the standard Euler-Maruyama method^53^. We construct the steady state fluctuations by

57 $${{{\mathcal{C}}}}_{ij}^{{{\rm{sto}}}}(\tau )={\overline{{s}_{i,1}^{(n)}(\tau ){s}_{j,2}^{(n)}(0)}}_{{{\rm{s.s.}}}}-{\overline{{s}_{i,1}^{(n)}}}_{{{\rm{s.s.}}}}\,{\overline{{s}_{j,2}^{(n)}}}_{{{\rm{s.s.}}}}\,,$$

where *τ* = *m*Δ*t* and ${\overline{(\cdot )}}_{s.s.}$ denotes an average over independent stochastic realizations (walkers) in the steady state. The simulation parameters used for Fig. 5 are: *Ω*_R_ = 1 unit scale, *Δ*/*Ω*_*R*_ = −0.08, 0, 0.08, *Ω*_R_*T*_1_ = 12, *Ω*_R_*T*_2_ = 2 ⋅ *T*_1_ and 4 × 10^3^.

### Experimental methods

Resonance fluorescence measurements were performed under resonant excitation of a single QD. The setup was designed to separate the reflected laser from the emission signal using cross-polarized filtering. The sample was cooled to 4.2 K in a liquid helium bath cryostat.

The sample was mounted on a piezoelectric positioning system, allowing 3D movement with 50 nm precision, and contacted via high-frequency connectors for voltage control of the Schottky diode. Excitation and collection were performed through a high-numerical-aperture (NA = 0.77) objective.

The optical head consisted of three stages: imaging, excitation, and detection. Excitation light, delivered via an optical fiber, was linearly polarized and reflected by a polarizing beamsplitter to illuminate the sample. Emitted resonance fluorescence photons were collected through the same objective, passed through the polarizing beamsplitter and additional polarizers, and coupled into a single-mode fiber for detection by a Fabry–Perot cavity. More precisely, the QD emission was detected with a scanning Fabry–Perot interferometer and a single-photon avalanche diode. A quarter-wave plate above the cryostat window corrected for polarization distortions, and the fiber coupling acted as a spatial filter to further suppress residual laser light.

## Supplementary information

Transparent Peer Review file

## Data Availability

The data generated in this study have been deposited in the Figshare database under accession codes 10.6084/m9.figshare.30893525 and 10.6084/m9.figshare.30893207.

## Code availability

The codes generated in this study have been deposited in the Zenodo database under accession code 10.5281/zenodo.19482744.
