# Supplementary material for: Bridging quantum noise and classical electrodynamics with stochastic methods
Source: Nat Commun. 2026 May 19;17:6618. doi: 10.1038/s41467-026-73066-4 (PMC13381871; doi:10.1038/s41467-026-73066-4)
Supplement: Supplementary file 1 — Transparent Peer Review file [file 41467_2026_73066_MOESM1_ESM.pdf]

nature portfolio

Peer Review File

Bridging Quantum Noise and Classical Electrodynamics with Stochastic Methods

Corresponding Author: Mr Felix Hitzelhammer

This file contains all reviewer reports in order by version, followed by all author rebuttals in order by version.

Version 0:

Reviewer comments:

Reviewer #1

(Remarks to the Author)
Many apparently quantum mechanical features of the electromagnetic field interacting with matter can be reproduced with classical physics plus a suitably chosen noise spectrum. In this work the authors extend this idea, illustrating their findings with an atom in a strong laser field exhibiting a Mollow triplet splitting and constructing a classical noise that effectively reproduces the correct quantum noise power spectrum S(\omega) computed from the correlation matrix. The novel step appears to be the use of a non-symmetric diffusion matrix, which is computed from Eq. (7) and then subject to a singular value decomposition (SVD). The two unitary matrices in the SVD are then used to construct *two* complex noise fields, which are understood as a vector valued stochastic process. This vector is then used to compute correlations that agree well with fully quantum mechanical calculations.

Overall I believe that this idea is an important novel contribution to the simulation of quantum systems. As I understand it, the core idea is present in Eqns. (7) and (9) and I have not seen this approach used before. It has the potential to extend earlier work to include the non-commutation of quantum operators in classical stochastic simulations.

However I have a number of questions concerning the results:

(1) Firstly something simple: I don't understand the normalization in Fig. 3. Why were the curves in Fig. 3 scaled to their respective maxima? Isn't there some normalization that can be used so that the classical and quantum results are identical without any such rescaling? In short, I want to check it simple to directly use the S(\omega) that comes out of this stochastic calculation, without any rescaling?

(2) Figure 4 shows the effect of measuring the power spectrum either at the atom, or using classical FDTD to propagate the field some distance away in (I think) vacuum. But does this really showcase the method? I think I could also do this fully quantum mechanically, as the Heisenberg equations of motion can be solved analytically for the field in terms of the initial state of the atom and field, which is here used to construct D. Then we can compute correlation functions of the field in terms of the initial density matrix. Could the authors explain why this particular example is used?

(3) Connected with (2), I think there should be a clearer statement of how the averaging was done with the FDTD simulations, how long is needed, and how this gives an advantage of a purely quantum mechanical treatment. For such quantum optics problems I usually think of the computational difficulty as being one of finding e.g. the cavity modes in a realistic system, which is a classical problem. There is also the difficulty of performing calculations when many degrees of freedom become entangled. Where does this method fit in here?

(4) Connected to (3). What are the general prospects here? Why focus on the Mollow triplet, what about other effects? If we imagine a future world where e.g. COMSOL or Lumerical have included a "stochastic quantum electromagnetism" module then who would use it, and for what? The first paragraph of the paper tries to address this point, but I found it was a bit non-specific. Presumably this method is not very useful for e.g. quantum computing or quantum communication, where we are dealing with entangled states?

(5) On a first read I found the presentation confusing. Eq. (1) is exactly the equation *not* being used here, as it is essential that D is not symmetric. It is not until Eq. (7) that we get introduced to the key idea. Perhaps a reordering of the text would

help here.

(6) A point on the general background. I think similar ideas to this go back a long way, before e.g. Drummond's work in the 1980s. There have been many attempts to reproduce quantum electrodynamic effects using classical theory plus noise. For example the theory of "Stochastic electrodynamics",

"Random Electrodynamics" T. W. Marshall Proc. Roy. Soc. A 276 (1963),

"Random electrodynamics: The theory of classical electrodynamics with classical electromagnetic zero-point radiation" T. Boyer Phys. Rev. D 11 (1975)

and Rytov's stochastic electrodynamic theory that was used as the basis for the modern theory of the Casimir effect,

"Theory of Electrical Fluctuations and Thermal Radiation." USSR: Publishing House, Russian Academy of Sciences. Rytov, S. M. (1953).

I know this work is slightly tangential to this paper, but it might help to give a slightly broader introduction that acknowledges some of this early work.

Reviewer #2

(Remarks to the Author)
The work by Hitzelgammer et al. presents a surprisingly successful attempt to develop a stochastic treatment for coherent quantum phenomena, focusing on the Mollow triplet, a foundational example of quantum light-matter interactions. The authors introduce a formalism based on 'classical' stochastic differential equations (SDEs) that, when augmented with appropriate assumptions regarding noise correlations, can effectively simulate this key quantum effect. A significant strength of this approach is its demonstrated capability to interface with standard Maxwell solvers, which could offer considerable advantages in the practical design of photonic structures, such as those incorporating semiconductor quantum dots (QDs). While this work is undeniably thought-provoking and interesting, offering a novel perspective on modeling coherent quantum dynamics, it is not without its weaknesses. Consequently, I recommend the manuscript for publication in Nature Communications, contingent upon the authors successfully addressing these identified limitations.

1. First and foremost, the authors suggest that their method offers computational advantages; however, this claim is not particularly well substantiated in its current form. The complexity of the SDE appears to be intimately linked to the rank of the SVD decomposition detailed in Eq. 8. Furthermore, as the proposed formalism is stochastic, it necessitates implementing multiple trajectories, which naturally incurs a computational cost, even if it allows for highly parallel implementation. The authors should state, in a quantitative manner, the actual cost of their algorithm versus the established quantum regression theorem.
2. Related to these comparisons, the authors also introduce a Green’s propagator approach. As far as can be determined, this is equivalent to the quantum regression theorem, merely written in a vectorised form. If this is the case, what unique value does this approach add to the manuscript?
3. The authors state that the method can be included consistently with FDTD (Finite-Difference Time-Domain) solvers; however, the photonic environment will naturally influence the light-matter coupling strength, specifically the spontaneous emission rate. This crucial effect does not appear to be included in the model, and would naturally introduce some kind of self-consistency (with an additional computational overhead, no doubt). Is the formalism truly capable of a consistent interface with FDTD?
4. Figure 7 displays the comparisons between experiment and theory; have the authors performed any fitting? Although the qualitative behavior is captured, the quantitative agreement seems quite poor.
5. Finally, the authors do not mention any of the macroscopic approaches for including photonic structures into quantum optics formalisms (e.g., Macroscopic Quantum Electrodynamics). My understanding is that MQED can indeed be interfaced with FDTD, what is the relation between these approaches?

Reviewer #3

(Remarks to the Author)
Hitzelhammer et al. develop a stochastic method for simulating a model of a light-matter system. The particular advantage that they cite this method as having over methods is that it can directly link to classical Maxwell solvers. These are useful in particular for dealing with propagating light modes. The modelling is focussed on describing the Mollow triplet, and indeed the simulations are compared to an experiment on a driven quantum dot.

The work is clearly described and well presented. However, I am unsure as to whether this work is an important enough advance for publication in Nature Communications. The particular advantage of coupling with Maxwell solvers, and why this is so important, is not clearly enough demonstrated in my opinion. If the authors were able to counter this, I would certainly consider the paper again.

I have a few more specific comments as follows:

1. P1, col 1, bottom: can the authors give an example of where the exponentially large Hilbert space is a key consideration?
2. P2, col 1: the authors here first mention sidebands appearing in the Mollow triplet. These sidebands are due to the

resonance optical driving; can further structure due to phonons be dealt with using this technique?
3. P2, Eq. 3: small thing - different format for sigma_z here and just before this equation.
4. P3,4: there is pretty much perfect agreement between the different panels in Figs. 2 and 3; the only difference is more noise in the stochastic plots. Why then, should I use the stochastic method rather than the others? I guess these plots are meant to show that the stochastic method works well; however, I would like to see a comparison of how long each simulation would take.
5. P6: more detail is needed around Eqs. 18 and 19. I am not sure all symbols are defined, and I think this is a crucial point of the paper. In addition, what causes the difference between the results in Fig. 4? This is a key figure I think and more insight into the differences would be helpful. Essentially, I am asking what happens during the propagation phase.
6. P7, Fig. 6: could these plots have been obtained using other techniques? Does the stochastic solver resolve any phonon sidebands?
7. P7, Fig. 6: what fixes the width of the peaks for the simulations here shown in Fig. 6(a)?
8. P8, Fig. 7: the comparison here is not great and this is addressed in the text. Would this be any different if other theoretical methods were used?
9. P9, conclusion. Is there a clear route to multi-time correlations here?
10. P10, Eq. 34: here, I assume that one (or possibly more) k value would have eigenvalue zero. Thus, could this equation be written in a way that highlights this more clearly?
11. More generally, the quantum regression theorem breaks down for non-Markovian dynamics, such as might be caused by phonons. Can this stochastic method deal with such non-Markovian effects?

Version 1:

Reviewer comments:

Reviewer #1

(Remarks to the Author)
The authors have responded to all of my previous points. I think the paper is now suitable for publication.

(Remarks on code availability)

Reviewer #3

(Remarks to the Author)

The authors have done a good job of responding to my comments in general. In particular, they do now articulate why this paper could lead to important advances in future. Though I would have liked to have seen a real computational advantage to be demonstrated in this paper, I think on balance I am happy to recommend publication.

Fig 4: I’m still worried about the disagreement between the methods here. Can the conjecture about non-point like sources be checked? Is it possible to vary the source size and get the agreement to be better or worse?

P10 and in the response, the authors talk about “the disbelief and reservations of the community.” Is this statement warranted? Can a reference be included to demonstrate this?

(Remarks on code availability)

Open Access This Peer Review File is licensed under a Creative Commons Attribution 4.0 International License, which permits use, sharing, adaptation, distribution and reproduction in any medium or format, as long as you give appropriate credit to the original author(s) and the source, provide a link to the Creative Commons license, and indicate if changes were made.
In cases where reviewers are anonymous, credit should be given to 'Anonymous Referee' and the source.
The images or other third party material in this Peer Review File are included in the article's Creative Commons license, unless indicated otherwise in a credit line to the material. If material is not included in the article's Creative Commons license and your intended use is not permitted by statutory regulation or exceeds the permitted use, you will need to obtain permission directly from the copyright holder.
To view a copy of this license, visit https://creativecommons.org/licenses/by/4.0/

Reply to Reviewers.

We thank all Reviewers for their thoughtful and constructive reports, and for pointing out where additional information is needed. Before addressing all specific questions and comments raised by the Reviewers in a point-to-point response, we would like to start by briefly emphasizing what we consider as the main novel contribution of our work.

For many years it has been thought that stochastic differential equations (SDEs) can only account for photon correlations of large ensembles of quantum emitters, see for instance the books of Carmichael. In our paper we show that, on the contrary, through a generalized non-symmetric diffusion matrix it is possible to reliably and robustly account for photon properties of a few or even a single quantum emitter. As described more explicitly in this revised manuscript, this establishes a link between quantum optics and the rich field of SDEs, and holds promise for numerous extensions including self-consistent light-matter interactions or non-Markovian time evolutions.

In combination with FDTD solvers, this could become a real game changer. It would allow us to simulate genuine quantum effects with classical Maxwell solvers. This is achieved by imprinting the quantum noise from the sources to the fields, which propagate in the same way as the classical ones. There is an overhead in computing time through the consideration of many simulations with different noise realizations (or a single, sufficiently long simulation in the steady state), but this could be acceptable considering that existing FDTD solvers could be used without introducing significant modifications, and the possibility of massive parallelization.

However, one can foresee scenarios where our approach is superior to existing ones even in its present form. A new generation of spectroscopy and microscopy techniques relies on photon correlation measurements for multiple emitters, harnessing all possible kinds of spatial, temporal, and spectral correlations (see e.g., the review G. Lubin et al., “Photon Correlations in Spectroscopy and Microscopy”, ACS Photonics 9, 2891-2904, 2022). In a traditional approach, one first has to characterize the field propagators for all possible source and field positions, as well as delay times, before convoluting them with the sources. In contrast, in our approach the quantum properties of the sources are transferred to the fields, and photon correlations can be directly computed from these simulated fields without establishing any explicit link to the sources. More details can be found in the revised manuscript.

The future will tell whether our envisioned combination of SDEs and FDTD is as fruitful as we are hoping for. In any case, we are convinced that our approach

makes an important step forward in methodology and conceptual developments, and we think that Nature Communications is the right platform to foster further studies and developments in this active field of emerging quantum technologies.

Response to the Reviewers' reports

– Our responses are written in blue color.

Report of the First Reviewer – Hitzelhammer

Many apparently quantum mechanical features of the electromagnetic field interacting with matter can be reproduced with classical physics plus a suitably chosen noise spectrum. In this work the authors extend this idea, illustrating their findings with an atom in a strong laser field exhibiting a Mollow triplet splitting and constructing a classical noise that effectively reproduces the correct quantum noise power spectrum $S(\omega)$ computed from the correlation matrix. The novel step appears to be the use of a non-symmetric diffusion matrix, which is computed from Eq. (7) and then subject to a singular value decomposition (SVD). The two unitary matrices in the SVD are then used to construct *two* complex noise fields, which are understood as a vector valued stochastic process. This vector is then used to compute correlations that agree well with fully quantum mechanical calculations.

Overall I believe that this idea is an important novel contribution to the simulation of quantum systems. As I understand it, the core idea is present in Eqns. (7) and (9) and I have not seen this approach used before. It has the potential to extend earlier work to include the non-commutation of quantum operators in classical stochastic simulations.

We are grateful to the Reviewer for the positive assessment and the insightful summary of our methodology developments.

Comment 1
Firstly something simple: I don't understand the normalization in Fig. 3. Why were the curves in Fig. 3 scaled to their respective maxima? Isn't there some normalization that can be used so that the classical and quantum results are identical without any such rescaling? In short, I want to check it simple to directly use the $S(\omega)$ that comes out of this stochastic calculation, without any rescaling?

The normalization of the respective spectra $S(\omega)$ to their own maxima was done for convenience and to keep all values between zero and one. Attached is Fig. 3(a)–(c) without normalization, where it can be seen that all three maxima are equal up to numerical errors.

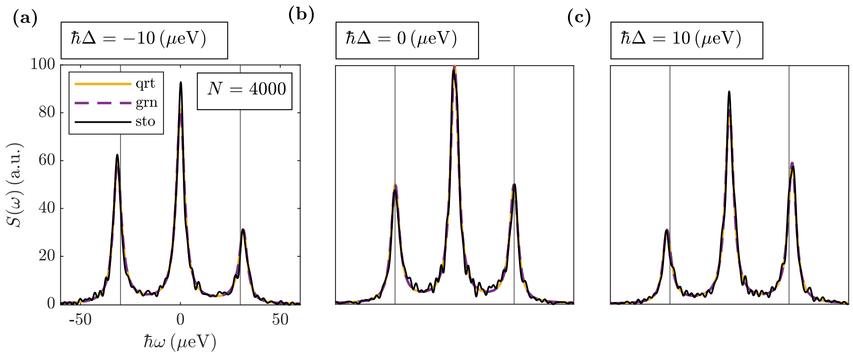

To prevent any misunderstandings, we have added this information to the caption of Fig. 3:

> "For convenience, the presented data is normalized to their respective maxima, which are equal up to numerical errors. Hence, this normalization does not introduce any distortions."

Comment 2

Figure 4 shows the effect of measuring the power spectrum either at the atom, or using classical FDTD to propagate the field some distance away in (I think) vacuum. But does this really showcase the method? I think I could also do this fully quantum mechanically, as the Heisenberg equations of motion can be solved analytically for the field in terms of the initial state of the atom and field, which is here used to construct D. Then we can compute correlation functions of the field in terms of the initial density matrix. Could the authors explain why this particular example is used?

To clarify that the FDTD simulations in our manuscript are performed in free space, we have added a sentence in the revised Results section "Stochastic Bloch vector coupled to an FDTD solver."

> "..., where ϵ is the dielectric function, which we set to unity to model a vacuum environment, Δt and Δx denote the time step and spatial grid spacing, respectively, and the superscript n and subscript i refer to the temporal and spatial indices of the FDTD grid."

We agree that, in principle, one could calculate the Mollow spectrum "at" the atom using a fully quantum-mechanical analytical solution. However, the point of this plot is to demonstrate that our approach provides direct access to spatial and temporal electric fields, which would be otherwise very difficult with the method mentioned by the Reviewer. In this way, we deal with exactly those quantities that are accessible in experiments, highlighting the practical impor-

tance of our coupling to an FDTD solver. To better explain these ideas, we have also added the following sentence at the end of the section "Stochastic Bloch vector coupled to an FDTD solver.

> "The direct access to spatial and temporal electric fields provided by our approach involving the FDTD method is desirable since we deal with exactly those quantities that are accessible in experiments."

Comment 3
Connected with (2), I think there should be a clearer statement of how the averaging was done with the FDTD simulations, how long is needed, and how this gives an advantage of a purely quantum mechanical treatment. For such quantum optics problems I usually think of the computational difficulty as being one of finding e.g. the cavity modes in a realistic system, which is a classical problem. There is also the difficulty of performing calculations when many degrees of freedom become entangled. Where does this method fit in here?

The averaging is performed by computing the ensemble average over stochastic realisations of the electric field. Numerically, the two-time correlation is estimated by evaluating the field at two different times within each realisation and subsequently averaging the corresponding products over an ensemble of statistically independent realisations. In terms of computational cost, this averaging step is not a limiting factor. We have added additional information in the revised Results section "Stochastic Bloch vector coupled to an FDTD solver".

> "..., where the overbar denotes an ensemble average over stochastic realisations of the electric field in the steady state. In practice, the two-time correlation is calculated by evaluating the positive- and negative-frequency field components at two times separated by τ within each realisation and averaging the resulting products over statistically independent realisations, see also Methods section. The incoherent contribution is obtained by subtracting the product of the corresponding steady-state mean fields."

Moreover, elaborating on the relevant introductory part of the response letter, we have added a new paragraph in the section "Discussion" which deals with the question of computational advantages.

> "From a computational perspective, our approach requires simulations for a sufficiently large number of stochastic noise realizations, which leads in general to significantly longer runtimes in comparison to more direct approaches. However, the major achievement of this work is the demonstration that genuine quantum effects can be modelled with classical FDTD solvers, despite the disbelief and reservations of the community. Considering that the approach holds potential for further improvements, such as the self-consistent treat-

ment of light-matter interactions discussed above, and the fact that classical FDTD solvers can be employed without any major modification, we think that the overhead in computer time is acceptable, in particular in view of the fast performance of FDTD simulations and the possibility of parallelization. However, one can also envision scenarios where our approach in its present form can already outperform more traditional approaches. A new generation of spectroscopy and microscopy techniques relies on photon correlation measurements for multiple emitters, harnessing all possible kinds of spatial, temporal, and spectral correlations [79]. In a traditional approach, one first has to characterize the field propagators for all possible source and field positions as well as delay times, before convoluting them with the sources. In contrast, in our approach the quantum properties of the sources are directly imprinted on the fields, and photon correlations can be computed from the simulated fields at different positions and times in a massively parallel fashion without explicitly establishing a link between sources and fields. In such setups our approach appears to be superior to the traditional approaches, both regarding conceptual simplicity and computational speed."

Comment 4
Connected to (3). What are the general prospects here? Why focus on the Mollow triplet, what about other effects? If we imagine a future world where e.g. COMSOL or Lumerical have included a "stochastic quantum electromagnetism" module then who would use it, and for what? The first paragraph of the paper tries to address this point, but I found it was a bit non-specific. Presumably this method is not very useful for e.g. quantum computing or quantum communication, where we are dealing with entangled states?

In order to validate our new theory and computational method, we are seeking for signatures of quantum light. One prominent example is resonance fluorescence and the Mollow triplet spectrum. We start with this particular quantum light signature as a proof of concept. Currently we are extending our approach to multi-time correlations, most importantly antibunching at zero-time delay in the second-order coherence function. A close-to-zero second-order coherence function is a measure of the emitted photon purity. High photon purity and indistinguishability are crucial for the realisation of quantum-optical gates in photonic quantum computing, quantum repeaters and optical quantum memories for secure quantum communications and quantum sensing technologies. Having a means to simulate such important photon properties with the aid of classical Maxwell solvers, such as COMSOL, would be of great importance for the modelling and optimization of integrated architectures on a chip. Since our vision was not sufficiently explained, we added the following paragraph to the section "Introduction" of the revised manuscript.

"The latter is of particular importance for design and optimisation

> of the key components of quantum-photonic technologies, e.g., quantum emitters, quantum memories and repeaters, etc. There is currently a lack of design, modeling and simulation tools for quantum photonics that would allow predicting and optimising the quantum performance of such realistic devices. Achieving peak quantum performance of the individual quantum-photonic components is paramount for realisation of the Quantum Photonic Integrated Circuits (QPICs) on a chip. Integrated architectures, and mass production of consistently highly-performant components, by common consent, represent the way forward to achieving scalability. A unified approach that bridges quantum and classical descriptions is therefore highly desirable not solely from a long-standing foundational point of view [13–15] but also for the practical realization of quantum technologies."

Regarding the Reviewer's statement of the utility of this approach for entangled states: quantum networks rely on entanglement distribution through quantum repeaters and entanglement swapping. Generation of high-quality single photons is indispensable for realization of quantum entanglement. Within our approach we can tackle both purity and indistinguishability of single photons (through optimization of the driving pulse characteristics and generation of a programmable single-photon waveform). Therefore, we believe our method will be useful for designing such quantum network nodes.

Comment 5
On a first read I found the presentation confusing. Eq. (1) is exactly the equation *not* being used here, as it is essential that D is not symmetric. It is not until Eq. (7) that we get introduced to the key idea. Perhaps a reordering of the text would help here.

We thank the Reviewer for the comment. We have added the following clarifying sentences at the beginning of the section "SDEs for symmetric vs. non-symmetric diffusion matrices" that we hope will prevent any confusion for the reader.

> "In the following, we introduce the concept of SDEs for non-symmetric diffusion matrices (see the central Eqs. (7) and (10)). To better motivate this innovation, we start by briefly summarizing the standard path from a (quantum) master equation to an SDE relying on an ordinary symmetric diffusion matrix (see Eqs. (1) and (2))."

Comment 6
A point on the general background. I think similar ideas to this go back a long way, before e.g. Drummond's work in the 1980s. There have been many attempts to reproduce quantum electrodynamic effects using classical theory plus noise. For example the theory of "Stochastic electrodynamics",

“Random Electrodynamics” T. W. Marshall Proc. Roy. Soc. A 276 (1963),

“Random electrodynamics: The theory of classical electrodynamics with classical electromagnetic zero-point radiation” T. Boyer Phys. Rev. D 11 (1975)

and Rytov’s stochastic electrodynamic theory that was used as the basis for the modern theory of the Casimir effect,

“Theory of Electrical Fluctuations and Thermal Radiation.” USSR: Publishing House, Russian Academy of Sciences. Rytov, S. M. (1953).

I know this work is slightly tangential to this paper, but it might help to give a slightly broader introduction that acknowledges some of this early work.

We think that the Reviewer raises an important point here. On the one hand, it demonstrates that the idea of describing quantum electrodynamics effects with classical fluctuating fields is surprisingly old. On the other hand, it could provide a possibility to describe light-matter interactions self-consistently. Currently, in our approach the noise imprinted from the sources accounts for the dynamics of the driven two-level system in presence of dephasing and relaxation rates. While the radiative decay rate can be conveniently obtained from supplementary simulations, a direct computation through noise fields, as suggested by the Reviewer, could allow for a self-consistent consideration of light-matter interaction. We have included an overview in the section “Discussion” of the revised manuscript, where we also cite the papers suggested by the Reviewer.

> “In our current approach we have introduced the relaxation and dephasing rates as parameters that have to be obtained from experiment or independent theoretical approaches. A particularly convenient one is the framework of macroscopic quantum electrodynamics [75], which avoids the explicit quantization of electromagnetic fields by introducing material noise terms, and allows to derive optical decay rates in a first-principles manner. Quite generally, in the weak coupling regime such rates could be easily obtained from supplementary FDTD simulations and introduced into our approach. In the strong-coupling regime, the mutual light-matter interaction has to be described explicitly [76]. Early work on Random Electrodynamics [77, 78] has shown that quantum effects in light-matter interactions can be mimicked through fluctuating classical fields, which has been used as the basis for the modern theory of the Casimir effect [78]. Although beyond the scope of this paper, we envision that within a related approach the radiative decay of quantum emitters could be directly accounted for in FDTD through the consideration of the back-action of the emitted random fields on the quantum emitters. Future work should also address possible non-Markovian contributions in the dynamics of the quantum emitters, see [20] for a detailed discussion.”

Report of the Second Reviewer – Hitzelhammer

The work by Hitzelgammer et al. presents a surprisingly successful attempt to develop a stochastic treatment for coherent quantum phenomena, focusing on the Mollow triplet, a foundational example of quantum light-matter interactions. The authors introduce a formalism based on 'classical' stochastic differential equations (SDEs) that, when augmented with appropriate assumptions regarding noise correlations, can effectively simulate this key quantum effect. A significant strength of this approach is its demonstrated capability to interface with standard Maxwell solvers, which could offer considerable advantages in the practical design of photonic structures, such as those incorporating semiconductor quantum dots (QDs). While this work is undeniably thought-provoking and interesting, offering a novel perspective on modeling coherent quantum dynamics, it is not without its weaknesses. Consequently, I recommend the manuscript for publication in Nature Communications, contingent upon the authors successfully addressing these identified limitations.

We thank the Reviewer for the precious time and appreciation of our paper.

Comment 1
First and foremost, the authors suggest that their method offers computational advantages; however, this claim is not particularly well substantiated in its current form. The complexity of the SDE appears to be intimately linked to the rank of the SVD decomposition detailed in Eq. 8. Furthermore, as the proposed formalism is stochastic, it necessitates implementing multiple trajectories, which naturally incurs a computational cost, even if it allows for highly parallel implementation. The authors should state, in a quantitative manner, the actual cost of their algorithm versus the established quantum regression theorem.

We thank the Reviewer for pointing out the issue regarding efficiency and computational time. The novel aspect of our work is the methodology development and the proof-of-principle simulations and in its present form the SDE solution is certainly slower in comparison to the quantum regression theorem and the evaluation of a field propagator. Typical runtimes are on the order of minutes for the SDE and seconds for the QRT on a normal desktop computer. However, as discussed at the beginning of our response letter, one can envision situations where the situation is different, such as the microscopy/spectroscopy example, which requires the investigation of photon correlations for multiple emitters. These questions are now addressed in more detail in a new paragraph in the section "Discussion":

> "From a computational perspective, our approach requires simulations for a sufficiently large number of stochastic noise realizations, which leads in general to significantly longer runtimes in comparison to more direct approaches. However, the major achievement of this work is the demonstration that genuine quantum effects can be modelled with classical FDTD solvers, despite the disbelief and

> reservations of the community. Considering that the approach holds potential for further improvements, such as the self-consistent treatment of light-matter interactions discussed above, and the fact that classical FDTD solvers can be employed without any major modification, we think that the overhead in computer time is acceptable, in particular in view of the fast performance of FDTD simulations and the possibility of parallelization. However, one can also envision scenarios where our approach in its present form can already outperform more traditional approaches. A new generation of spectroscopy and microscopy techniques relies on photon correlation measurements for multiple emitters, harnessing all possible kinds of spatial, temporal, and spectral correlations [79]. In a traditional approach, one first has to characterize the field propagators for all possible source and field positions as well as delay times, before convoluting them with the sources. In contrast, in our approach the quantum properties of the sources are directly imprinted on the fields, and photon correlations can be computed from the simulated fields at different positions and times in a massively parallel fashion without explicitly establishing a link between sources and fields. In such setups our approach appears to be superior to the traditional approaches, both regarding conceptual simplicity and computational speed."

Moreover, since the use of an SVD is not strictly needed and one could resort to alternative, potentially computationally faster algorithms such as matrix square roots, we have also added the following sentence to the section "Discussion":

> "In this work we have employed SVDs for the factorization, but one could also use other schemes, such as matrix square roots, which might be beneficial for large matrices in order to speed up the simulations."

Comment 2
Related to these comparisons, the authors also introduce a Green's propagator approach. As far as can be determined, this is equivalent to the quantum regression theorem, merely written in a vectorised form. If this is the case, what unique value does this approach add to the manuscript?

The Green's propagator approach serves mainly as a consistency check regarding the SDEs, as the Green's propagator approach directly involves the drift matrix $\mathbf{A}$, i.e., the deterministic part of the SDE, rather than the full Liouvillian. We have expanded the last sentence in the section "Projection of $\mathcal{L}^\dagger$" to better highlight its role in the manuscript.

> "..., where the Green's propagator method is primarily used as a consistency check for benchmarking our stochastic approach since it directly involves the drift matrix $\mathbf{A}$, i.e., the deterministic part of

the SDE, rather than the full Liouvillian."

Comment 3
The authors state that the method can be included consistently with FDTD (Finite-Difference Time-Domain) solvers; however, the photonic environment will naturally influence the light-matter coupling strength, specifically the spontaneous emission rate. This crucial effect does not appear to be included in the model, and would naturally introduce some kind of self-consistency (with an additional computational overhead, no doubt). Is the formalism truly capable of a consistent interface with FDTD?

We thank the Reviewer for bringing up this point, which is also raised by the first Reviewer. Currently we treat the radiative decay rate as a parameter that has to be obtained from either experiment or supplementary simulations. Conveniently, the Purcell enhancement of the decay rate can be related to the imaginary part of the Green's tensor, with the source and field positions set equal to the position of the quantum emitter. Quite generally, this quantity can be directly obtained from additional FDTD simulations. As brought to our attention by the first Reviewer, there exists earlier work on stochastic electrodynamics that shows how to compute the light-matter interaction using classical fluctuating fields in a self-consistent manner. Although beyond the scope of this paper, this could be an interesting route of investigation for the future. In the section "Discussion" of the revised manuscript, we have now added a paragraph where all these points are addressed in some length (see our answer to the related Comment 5).

Comment 4
Figure 7 displays the comparisons between experiment and theory; have the authors performed any fitting? Although the qualitative behavior is captured, the quantitative agreement seems quite poor.

The only fitting performed is by approximating the individual peaks of the Mollow triplet by single Lorentzians, before integrating the respective area under the peaks. Overall, we observe good qualitative agreement between simulations and experiment in Fig. 6 and Fig. 7. Small discrepancies may arise from experimental limitations, including fluctuations and drifts in laser power and detuning, finite spectral resolution, laser-induced heating, and a wavelength-dependent detection efficiency caused by cross-polarized suppression of the excitation laser. The most pronounced deviation is observed in Fig. 7 when quantitatively comparing the intensity as a function of laser detuning. This discrepancy may be attributed to spectral diffusion of the quantum emitter as well as phonon-related effects beyond pure dephasing, which are not included in the model. As summarized in the following new sentence in the section "Emission spectrum of an InGaAs quantum dot", a part of the mismatch in Fig. 6 and 7 is due to experimental imperfections.

"The observed small differences between simulation and experiment

in Fig. 6 and Fig. 7 can be attributed to experimental inaccuracies such as fluctuations and drifts in power and detuning, limited spectral resolution, laser induced heating, and a spectral dependence of the efficiency resulting from the cross-polarized suppression of the excitation laser."

Comment 5

Finally, the authors do not mention any of the macroscopic approaches for including photonic structures into quantum optics formalisms (e.g., Macroscopic Quantum Electrodynamics). My understanding is that MQED can indeed be interfaced with FDTD, what is the relation between these approaches?

This is an interesting question raised by the Reviewer. To the best of our understanding, MQED can be used to compute the optical decay rates and the SDEs to compute photon correlations. MQED generally assumes a linear photonic environment, and avoids the explicit quantization of the electromagnetic fields by introducing quantum source terms for the material response together with the use of the classical electromagnetic Green's function for the photon propagation. By tracing over the source terms (environment) and using the fluctuation-dissipation theorem, one obtains expressions for the decay rates in a first-principles manner. Quite generally, our approach builds on this by using such decay rates together with the classical propagation properties of photons.

However, the photon correlations depend on the noise properties of the quantum emitter, which include both the coherent and incoherent quantum dynamics. These additional noise contributions (on top of the radiative decay rate) are modelled through our SDE framework. So MQED and SDE work together and account for the different physical processes entering our approach, although they could be probably brought even closer to each other through the self-consistency described above. The following detailed discussion of these points has been added to the section "Discussion" of the revised manuscript.

> "In our current approach we have introduced the relaxation and dephasing rates as parameters that have to be obtained from experiment or independent theoretical approaches. A particularly convenient one is the framework of macroscopic quantum electrodynamics [75], which avoids the explicit quantization of electromagnetic fields by introducing material noise terms, and allows to derive optical decay rates in a first-principles manner. Quite generally, in the weak coupling regime such rates could be easily obtained from supplementary FDTD simulations and introduced into our approach. In the strong-coupling regime, the mutual light-matter interaction has to be described explicitly [76]. Early work on Random Electrodynamics [77, 78] has shown that quantum effects in light-matter interactions can be mimicked through fluctuating classical fields, which has been used as the basis for the modern theory of the Casimir effect [78]. Although beyond the scope of this paper, we envision that

> within a related approach the radiative decay of quantum emitters could be directly accounted for in FDTD through the consideration of the back-action of the emitted random fields on the quantum emitters. Future work should also address possible non-Markovian contributions in the dynamics of the quantum emitters, see [20] for a detailed discussion."

Report of the Third Reviewer – Hitzelhammer

Hitzelhammer et al. develop a stochastic method for simulating a model of a light-matter system. The particular advantage that they cite this method as having over methods is that it can directly link to classical Maxwell solvers. These are useful in particular for dealing with propagating light modes. The modelling is focussed on describing the Mollow triplet, and indeed the simulations are compared to an experiment on a driven quantum dot.

The work is clearly described and well presented. However, I am unsure as to whether this work is an important enough advance for publication in Nature Communications. The particular advantage of coupling with Maxwell solvers, and why this is so important, is not clearly enough demonstrated in my opinion. If the authors were able to counter this, I would certainly consider the paper again.

We thank the Reviewer for the assessment. At the beginning of this response letter, we have summarized our main reasons why we believe that our work merits publication in Nature Communications, particularly in view of the long history of the subject and the many unsuccessful attempts to extend SDEs to single quantum emitters. Indeed, we believe that our contribution makes an important step forward and could stimulate numerous follow-up investigations. The prospect of simulating quantum effects with the aid of conventional classical Maxwell solvers would be a complete game changer, considering both the huge success and the widespread availability of FDTD solvers, as well as the tremendous importance of addressing quantum effects in quantum optical setups. Although our work is a proof-of-principle, we are thus convinced that its impact is far-reaching.

Comment 1
P1, col 1, bottom: can the authors give an example of where the exponentially large Hilbert space is a key consideration?

We have expanded the relevant paragraph in the section "Introduction" to clarify in which context the exponentially large Hilbert space becomes relevant.

> "Quantum descriptions such as the Gorini–Kossakowski–Sudarshan–Lindblad formalism [16, 17] or quantum stochastic differential equations [18] are elegant and fundamental. However, they do not provide access to the spatial and temporal properties of the electromagnetic field, nor do they offer a natural route for coupling to classical Maxwell

> solvers. One could instead rely on quantum electrodynamics methods, which quantize the electromagnetic field, but these approaches can quickly become computationally prohibitive for large or complex systems due to the exponential growth of the Hilbert space [19, 20]."

Here the newly added reference C. Schäfer et al., "Making ab initio QED functional(s): Nonperturbative and photon-free effective frameworks for strong light–matter coupling", PNAS 118, e2110464118, 2021 ([19] in the revised manuscript) supports our claim as can be seen from the direct quote:

> *"Detailed theoretical explanations are largely missing, as strong light–matter coupling in principle calls for investigations within the framework of quantum electrodynamics (QED). This, however, leads to a substantial increase in computational complexity, since the combined light–matter Hilbert space becomes prohibitively large."*

Comment 2

P2, col 1: the authors here first mention sidebands appearing in the Mollow triplet. These sidebands are due to the resonance optical driving; can further structure due to phonons be dealt with using this technique?

The approach in its current form does not account explicitly for phonon interactions. We include in our simulations a genuine pure dephasing term in the Lindblad equation, which accounts for broadening effects in the Mollow triplet, but it does not give rise to phonon sidebands. We have tried to account for phonon-induced effects following the approach in Refs. [R1, R2], but this analysis has not led to significant improvements. To provide a more complete description of our work, we have added the following paragraph to the section "Emission spectrum of an InGaAs quantum dot":

> "Here we note that we also attempted to account for phonon effects in the Markovian regime [64–66] by modifying the dephasing rate of the respective Lindblad jump operator, which did not lead to a significantly improved agreement."

A detailed description of phonon dephasing, as presented e.g. in [R3], is beyond the scope of this paper, but a possible solution could be to extend the computational space and to introduce a few representative phonon modes.

[R1] A. J. Ramsay et al., Effect of detuning on the phonon induced dephasing of optically driven InGaAs/GaAs quantum dots. J. Appl. Phys., 109, 102415 (2011).
[R2] Y.-M. He et al., Coherently driving a single quantum two-level system with dichromatic laser pulses. Nat. Phys., 15, 941-946 (2019).
[R3] K. Boos et al., Signatures of Dynamically Dressed States. Phys. Rev. Lett. 132, 053602 (2024).

Comment 3
P2, Eq. 3: small thing - different format for σ_z here and just before this equation.

We thank the Reviewer for spotting the typo, and have corrected it in the revised manuscript.

Comment 4
P3,4: there is pretty much perfect agreement between the different panels in Figs. 2 and 3; the only difference is more noise in the stochastic plots. Why then, should I use the stochastic method rather than the others? I guess these plots are meant to show that the stochastic method works well; however, I would like to see a comparison of how long each simulation would take.

If one is interested in modeling the emission spectrum using atom operators only, then the Green's propagator and the quantum regression theorem provide a faster approach. As pointed out by the Reviewer, Figures 2 and 3 serve as benchmarks: Figure 2 demonstrates convergence to the 'true' solution, and Figure 3 benchmarks the stochastic approach against established techniques. At the same time, these figures also illustrate that a new methodology is available, one in which allowing a non-symmetric diffusion matrix in the derivation of the SDEs represents a departure from existing approaches. The stochastic approach truly begins to shine when we couple these now 'classical' stochastic trajectories to FDTD solvers (Fig. 4). This allows us to incorporate classical electric fields that capture the full quantum correlations. To address these important questions, we have added a new paragraph in the section "Discussion":

> "From a computational perspective, our approach requires simulations for a sufficiently large number of stochastic noise realizations, which leads in general to significantly longer runtimes in comparison to more direct approaches. However, the major achievement of this work is the demonstration that genuine quantum effects can be modelled with classical FDTD solvers, despite the disbelief and reservations of the community. Considering that the approach holds potential for further improvements, such as the self-consistent treatment of light-matter interactions discussed above, and the fact that classical FDTD solvers can be employed without any major modification, we think that the overhead in computer time is acceptable, in particular in view of the fast performance of FDTD simulations and the possibility of parallelization. However, one can also envision scenarios where our approach in its present form can already outperform more traditional approaches. A new generation of spectroscopy and microscopy techniques relies on photon correlation measurements for multiple emitters, harnessing all possible kinds of spatial, temporal, and spectral correlations [79]. In a traditional approach, one first has to characterize the field propagators for all possible source and field positions as well as delay times, before convoluting them with the

sources. In contrast, in our approach the quantum properties of the sources are directly imprinted on the fields, and photon correlations can be computed from the simulated fields at different positions and times in a massively parallel fashion without explicitly establishing a link between sources and fields. In such setups our approach appears to be superior to the traditional approaches, both regarding conceptual simplicity and computational speed."

Comment 5

P6: more detail is needed around Eqs. 18 and 19. I am not sure all symbols are defined, and I think this is a crucial point of the paper. In addition, what causes the difference between the results in Fig. 4? This is a key figure I think and more insight into the differences would be helpful. Essentially, I am asking what happens during the propagation phase.

We are thankful for the close analysis and agree with the Reviewer that some symbols were not properly defined. We have added the following clarifying statements in the section "Stochastic Bloch vector coupled to an FDTD solver" of the revised manuscript.

> "We propagate the positive and negative frequency (denoted $\pm$) electric and magnetic field components by [63]
>
> $$\mathscr{E}_y^{(\pm)}\Big|_i^{n+1} = \mathscr{E}_y^{(\pm)}\Big|_i^{n} - \frac{\Delta t}{\varepsilon \Delta x}\left(\mathscr{H}_z^{(\pm)}\Big|_{i+\frac{1}{2}}^{n+\frac{1}{2}} - \mathscr{H}_z^{(\pm)}\Big|_{i-\frac{1}{2}}^{n+\frac{1}{2}}\right) - \frac{\Delta t}{\varepsilon}\,\mathscr{J}_{\text{source}}^{(\pm)}\Big|_i^{n+\frac{1}{2}}, \qquad (18)$$
>
> $$\mathscr{H}_z^{(\pm)}\Big|_{i+\frac{1}{2}}^{n+\frac{1}{2}} = \mathscr{H}_z^{(\pm)}\Big|_{i+\frac{1}{2}}^{n-\frac{1}{2}} - \frac{\Delta t}{\Delta x}\left(\mathscr{E}_y^{(\pm)}\Big|_{i+1}^{n} - \mathscr{E}_y^{(\pm)}\Big|_{i}^{n}\right), \qquad (19)$$
>
> where ε is the dielectric function, which we set to unity to model a vacuum environment, Δt and Δx denote the time step and spatial grid spacing, respectively, and the superscript n and subscript i refer to the temporal and spatial indices of the FDTD grid. In summary, the TLS radiates into the classical grid through the source current $\mathscr{J}_{\text{source}}^{\pm}(x,t)$."

Regarding the question of what causes the difference in Fig. 4, the most likely explanation is the spreading of the source current over several grid cells. Here the Gaussian envelope effectively replaces the quantum point dipole with an extended emitter, which reduces the effective field at the emitter's position and leads to smaller Mollow sideband amplitudes in the FDTD spectrum.

Comment 6

P7, Fig. 6: could these plots have been obtained using other techniques? Does the stochastic solver resolve any phonon sidebands?

Yes, these plots could also have been obtained using other techniques, such as the Green's propagator or the quantum regression theorem, but not with better agreement, apart from the noise visible on top of the solid lines in Fig. 6(a) and Fig. 7(a). However, this noise could be reduced simply by increasing the number of walkers. All three methods rely on similar assumptions, such as Markovianity. The stochastic solver does not resolve any phonon sidebands, for this, we refer to the extended discussion of dephasing and phonons in our framework provided in Comment 2.

Comment 7
P7, Fig. 6: what fixes the width of the peaks for the simulations here shown in Fig. 6(a)?

We are not entirely sure how to understand the question correctly. The width of the peaks in the Mollow spectrum is determined by the relaxation rate Γ_1 and the dephasing rate Γ_2. For each Rabi energy, the peaks of both the simulated and experimental Mollow spectra were fitted with Lorentzians, and the areas under these curves were then integrated and plotted as a function of the Rabi energy.

Comment 8
P8, Fig. 7: the comparison here is not great and this is addressed in the text. Would this be any different if other theoretical methods were used?

Regarding the comparison of experiment and simulation: Overall, we observe good qualitative agreement between simulations and experiment in Fig. 6 and Fig. 7. Small differences can occur due to experimental inaccuracies such as fluctuations and drifts in power and detuning, limited spectral resolution, laser induced heating, and a spectral dependence of the efficiency resulting from the cross-polarized suppression of the excitation laser. The largest difference is visible in Fig. 7 when quantitatively comparing the intensity as a function of the laser detuning. This may result from spectral diffusion of the quantum emitter and phonon effects beyond pure dephasing which are not taken into account. As summarized in the following new sentence in the section "Emission spectrum of an InGaAs quantum dot", a part of the mismatch in Fig. 6 and 7 is due to experimental imperfections.

> "The observed small differences between simulation and experiment in Fig. 6 and Fig. 7 can be attributed to experimental inaccuracies such as fluctuations and drifts in power and detuning, limited spectral resolution, laser induced heating, and a spectral dependence of the efficiency resulting from the cross-polarized suppression of the excitation laser."

Several established theoretical frameworks exist for treating phonon effects in more detail, such as the polaron master equation or the variational polaron master equation, but these lie beyond the scope of the present paper. For

reference we have also added the following sentence to the section "Emission spectrum of an InGaAs quantum dot".

> "To what extent the comparison of simulation and experiment can be improved using more advanced methods, such as the polaron master equation or variational polaron master equation [67, 68], lies beyond the scope of this work."

Comment 9
P9, conclusion. Is there a clear route to multi-time correlations here?

We think that this question can be answered affirmatively. The key should be to generalize the process that led us to noise matrices in Eq. (10) by way of the equation of motion for the covariance matrix, see Eq. (46). We have started to develop the methodology along these lines with promising but not yet mature results. The following paragraph in the section "Discussion" of the revised manuscript provides an outlook regarding this important next step.

> "In this work we have determined the noise matrices in Eq. (10) such that they lead to the proper equation of motion for the covariance matrix, see Eq. (46). We can speculate that along the same lines one could introduce higher-order noise contributions [74] that have to be chosen such that they lead to the corresponding equations of motion for the higher-order correlation matrices, although further work is needed to work out the details."

Comment 10
P10, Eq. 34: here, I assume that one (or possibly more) k value would have eigenvalue zero. Thus, could this equation be written in a way that highlights this more clearly?

We thank the Reviewer for pointing out this ambiguity. We have revised the steady-state expression in Eq. (34) to explicitly show that only the Liouvillian eigenmodes with $\mathrm{Re}\{\lambda_k\} = 0$ contribute in the long-time limit since modes with $\mathrm{Re}\{\lambda_k\} < 0$ decay exponentially as $t \to \infty$. For the parameter ranges considered in this work, the Liouvillian has a single eigenvalue $\lambda_0 = 0$, so the steady state is unique. This part of the section "Second-order cumulant matrix" now reads:

> "We obtain the steady-state solutions in the long-time limit $|\rho_{\mathrm{s.s.}}\rangle\rangle := \lim_{t\to\infty} |\rho(t)\rangle\rangle$ via
>
> $$|\rho_{\mathrm{s.s.}}\rangle\rangle = \sum_{\substack{k \\ \mathrm{Re}\{\lambda_k\}=0}} |r_k\rangle\rangle\langle\langle l_k|\sigma_{\mathrm{gg}}\rangle\rangle \,, \tag{34}$$
>
> where the ground-state supervector $|\sigma_{\mathrm{gg}}\rangle\rangle$, see Table II, is used as an initial state. Here we have used that terms with $\mathrm{Re}\{\lambda_k\} < 0$ decay as $t \to \infty$ and therefore do not contribute to the steady state."

Comment 11
More generally, the quantum regression theorem breaks down for non-Markovian dynamics, such as might be caused by phonons. Can this stochastic method deal with such non-Markovian effects?

As stated in our response to Comment 2, the effects of phonon-induced dephasing are currently captured by a single parameter, $\sqrt{\Gamma_2}$, which enters our approach through the Lindblad operators. More detailed modeling of phonon-induced effects has not shown a significant advantage when compared with the emission spectrum of the InGaAs quantum dot. Since the Lindblad equation, in its current form, inherently assumes Markovian dynamics, we cannot yet conclude whether such effects can be fully incorporated. It remains an interesting question whether classical SDEs can be reformulated to describe non-Markovian regimes, and we would like to address this in a future work.

Reply to Reviewers.

We are grateful to the Reviewers for another round of thoughtful and constructive feedback. Revisiting the manuscript in light of their comments has led to further improvements. Please find below our point-to-point responses to all raised questions and comments. The changes made in the manuscript are highlighted in green.

Response to the Reviewers' reports

– Our responses are written in blue color.

Report of the Third Reviewer – NCOMMS-25-77307A/Hitzelhammer

The authors have done a good job of responding to my comments in general. In particular, they do now articulate why this paper could lead to important advances in future. Though I would have liked to have seen a real computational advantage to be demonstrated in this paper, I think on balance I am happy to recommend publication.

Comment 1

Fig 4: I'm still worried about the disagreement between the methods here. Can the conjecture about non-point like sources be checked? Is it possible to vary the source size and get the agreement to be better or worse?

We thank the Reviewer for insisting to improve the agreement between the spectra with and without consideration of propagation effects in FDTD. In Fig. 4 of the revised manuscript we now obtain much better agreement by averaging the two-time correlation over all starting times t, rather than evaluating it at a single reference point $t = 0$, as we had previously done. In principle, we believe that the agreement could be even further increased by introducing a number of minor computational improvements. However, this would not modify the main conclusions of our work and we thus have refrained from further elaborating on this point here. Attached is the reworked Fig.4 (a–c).

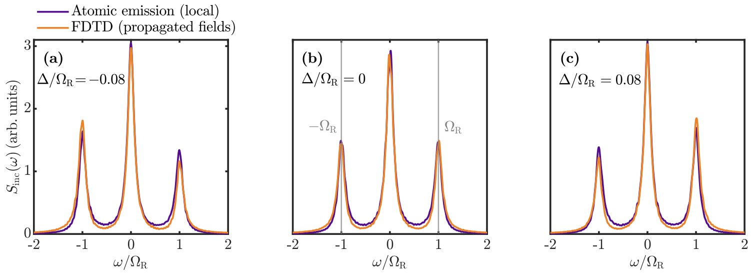

To reflect this improvement we have altered the relevant sentence in the revised Results section "Stochastic Bloch vector coupled to a FDTD solver":

> "In practice, the two-time correlation is calculated by evaluating the positive- and negative-frequency field components at two times separated by τ within each realisation and averaging the resulting products over all starting times and statistically independent realisations, see also Methods section."

Comment 2

P10 and in the response, the authors talk about "the disbelief and reservations of the community." Is this statement warranted? Can a reference be included to demonstrate this?

With this statement, we refer to Prof. Carmichael's comprehensive work on Statistical Methods in Quantum Optics. We summarize the limitation (Vol. 1, Ch. 6) to which we refer as follows: Within the framework of Chapter 6 dealing with the quantum-classical correspondence, the single two-level atom is essentially a limiting case in which the correspondence fails, and no alternative dynamical-stochastic picture is offered at this point. Although Carmichael later points to an alternative approach using quantum-stochastic trajectories, this involves evolving a quantum state and is a completely different conceptual framework. As a clarification we have added the following new sentence in the section "Discussion":

> "For example, a possible caveat is that the quantum-classical correspondence is believed to fail for certain limiting cases such as the single two-level atom [14]."
